# Relationship between parity and the prevalence of chronic kidney disease in Japan considering hypertensive disorders of pregnancy and body mass index

**DOI:** 10.1186/s12882-024-03604-z

**Published:** 2024-05-17

**Authors:** Hongxin Wang, Noriyuki Iwama, Keiichi Yuwaki, You Nakamichi, Hirotaka Hamada, Hasumi Tomita, Kazuma Tagami, Rie Kudo, Natsumi Kumagai, Hirohito Metoki, Naoki Nakaya, Atsushi Hozawa, Shinichi Kuriyama, Nobuo Yaegashi, Masatoshi Saito

**Affiliations:** 1 Department of Obstetrics and Gynecology, Tohoku University Hospital, 1-1, Seiryomachi, Sendai, Miyagi 980-8574 Japan; 2 Women’s Health Care Medical Science, Tohoku University Graduate School of Medicine, 1-1, Seiryomachi, Sendai, Miyagi 980-8574 Japan; 3 Tohoku Medical Megabank Organization, Tohoku University, 2-1, Seiryomachi, Sendai, Miyagi 980-8573 Japan; 4 Underwriting and Medical Department, The Dai-ichi Life Insurance Company, Limited, Koto-ku, Tokyo, Japan; 5 Division of Public Health, Hygiene and Epidemiology, Tohoku Medical Pharmaceutical University, 1-15-1 Fukumuro, Sendai, Miyagi 983-8536 Japan; 6 Division of Molecular Epidemiology, Tohoku University Graduate School of Medicine, 1-1, Seiryomachi, Sendai, Miyagi 980-8574 Japan; 7 International Research Institute of Disaster Science, Tohoku University, 468-1, Aramaki, Sendai, Miyagi 980-8572 Japan; 8 Environment and Genome Research Center, Tohoku University Graduate School of Medicine, 2-1, Seiryomachi, Sendai, Japan Sendai, Miyagi 980-8575 Japan; 9 Department of Maternal and Fetal Therapeutics, Tohoku University Graduate School of Medicine, 1-1, Seiryomachi, Sendai, Miyagi 980-8574 Japan

**Keywords:** Parity, Body mass index, Chronic kidney disease, Hypertensive disorders of pregnancy

## Abstract

**Background:**

Global studies exploring the relationship between parity and chronic kidney disease (CKD) are scarce. Furthermore, no study has examined the relationship between parity and CKD in Japan. Therefore, this study aimed to examine the relationship between parity and the prevalence of CKD in a Japanese population, considering the clinical history of hypertensive disorders of pregnancy (HDP) and current body mass index (BMI) based on menopausal status.

**Methods:**

This cross-sectional study included 26,945 Japanese multiparous women (5,006 premenopausal and 21,939 postmenopausal women) and 3,247 nulliparous women (1,599 premenopausal and 1,648 postmenopausal women). Participants were divided into two groups based on their menopausal status (premenopausal and postmenopausal women). The relationship between parity and the prevalence of CKD was evaluated using a multiple logistic regression model adjusted for several covariates, including a clinical history of HDP and current BMI.

**Results:**

The relationship between parity and the prevalence of CKD was not statistically significant in either premenopausal or postmenopausal multiparous women. A clinical history of HDP was significantly associated with an increased risk of CKD in premenopausal and postmenopausal multiparous women. However, the relationship between a clinical history of HDP and CKD in premenopausal women was weakened after adjusting for current BMI. Furthermore, the current BMI was significantly associated with an increased risk of CKD in both premenopausal and postmenopausal women.

**Conclusions:**

Parity is not significantly associated with the prevalence of CKD in premenopausal and postmenopausal multiparous women. A clinical history of HDP is a risk factor for CKD in both premenopausal and postmenopausal women. Current BMI is also associated with an increased risk of CKD in premenopausal and postmenopausal women. Therefore, continuous surveillance and preventive measures against CKD should be provided to women with a clinical history of HDP. In addition, maintaining an appropriate body weight is beneficial in reducing the risk of CKD.

**Supplementary Information:**

The online version contains supplementary material available at 10.1186/s12882-024-03604-z.

## Background

Chronic Kidney Disease (CKD) is an escalating global health concern marked by its increased prevalence over the past few decades [1]. CKD affects 8–16% of the global population and has substantially affected public health and healthcare economies [2]. Patients with stage 5 CKD or end-stage renal disease (ESRD) often require dialysis or kidney transplantation, which further exacerbates the global medical and economic burden [3].

Japan particularly faces a challenge because it has the highest reported global prevalence of ESRD [4]. Therefore, implementing measures to prevent CKD in the Japanese population is essential. The two primary causes of CKD and well-established global risk factors are type 2 diabetes mellitus (T2DM) and hypertension [5, 6]. Notably, numerous epidemiological studies have explored the relationship between parity and women’s health in their later years [7–11]. Higher parity has been associated with an increased prevalence of CKD in middle-aged and elderly Chinese women, highlighting the potential influence of reproductive history on kidney health [7]. Among Iranian women, higher parity was associated with a higher risk of incident hypertension, increasing the growing body of evidence connecting parity to cardiovascular health [8]. Previous studies have shown a linear-graded relationship between higher parity and the risk of T2DM [9]. Furthermore, parity has been reported as associated with obesity [10, 11], indicating that reproductive history may have more consequences on women’s health.

Women who experience hypertensive disorders of pregnancy (HDP), a specific risk factor for hypertension in women, are also reported to have an elevated risk of developing CKD later in life compared with those without a history of HDP, highlighting the long-term effects of pregnancy complications on kidney health [12].

Previous studies have revealed intriguing relationships between parity and various health outcomes; however, studies exploring the relationship between parity and CKD are scarce globally. Furthermore, no study has examined the relationship between parity and CKD in Japan. Obesity is an established risk factor of CKD [2]. Japanese women have a significantly lower body mass index (BMI) than Western women, and different lifestyles suggest that the relationship between parity and the risk of CKD may differ between Japanese women and women in other countries [13, 14].

Therefore, this study aimed to clarify the relationship between parity and the prevalence of CKD in Japan. We considered HDP and BMI and underscored their importance in our research based on its pronounced impact on women’s long-term health.

## Methods

### Study design and participants

This cross-sectional study used data from a type 1 survey conducted by the Tohoku Medical Megabank Community-based Cohort Study (TMM CommCohort Study). This prospective cohort study was initiated in 2013 and is ongoing in Miyagi and Iwate prefectures of Japan. The TMM CommCohort Study was established following the Great East Japan Earthquake (GEJE) and the subsequent tsunami that caused severe damage along the Pacific coast of the Tohoku region in 2011, as previously described [15, 16], aims to contribute to post-disaster recovery efforts and address medical concerns. The TMM CommCohort Study enrolled both men and women; however, the present study included only women who met the following criteria: (1) age ≥20 and <75 years and residing in the Miyagi or Iwate prefectures during the baseline survey conducted between May 2013 and March 2016, and (2) provided written informed consent to participate in the study during the municipal health checkup. This study was approved by the Institutional Review Board of the Tohoku University School of Medicine (approval numbers 2021-1-608, 2022-1-069, and 2022-1-216).

In total, 40,712 women who fulfilled the inclusion criteria were included in this study. We stratified the study participants into premenopausal and postmenopausal groups because most women with ESRD are in the postmenopausal age group [17], and fertility potential differs based on menopausal status.

## Data collection

### Parity

Information regarding the number of children was acquired using self-reported questionnaires. Parity, highlighted as this study’s exposure of interest, was characterized by the number of children and grouped as nulliparous (parity = 0), 1, 2, 3, and ≥4. Notably, we did not collect data on stillbirths or multiple pregnancies.

### Definition of CKD in this study

The study’s outcome was CKD. Venous blood and urine samples were collected from the municipal health checkup venues. The participants were diagnosed with CKD if they met any of the following criteria: (1) Urine albumin-to-creatinine ratio (ACR) ≥ 30 mg/gCre; (2) Estimated glomerular filtration rate (eGFR) < 60 mL/min/1.73m^2^ [1].　Urine microalbumin and creatinine levels were measured using quantitative immunoturbidimetry and enzymatic assays, respectively [16]. eGFR was calculated using the following formula: (104 × serum cystatin C (CysC)^–1.019^ × 0.996^age^ × 0.929) – 8 [18]. Serum cystatin C levels were measured using latex agglutination turbidimetry [16].

### Clinical history of HDP

A clinical history of HDP was obtained using a self-reported questionnaire in response to the question, “Have you ever been diagnosed with hypertensive disorders of pregnancy or toxemia?”[19].

### Clinical history of GDM

Gestational diabetes (GDM) was diagnosed based on the 1984 Japan Society of Obstetrics and Gynecology criteria [20]. A clinical history of GDM was obtained using a self-report questionnaire in response to the question, “Have you ever been diagnosed with gestational diabetes mellitus?”.

### Definition of premenopausal and postmenopausal women

Premenopausal and postmenopausal women were categorized based on their responses to a self-reported questionnaire regarding their current menstrual status. Participants were asked to select one of the three options: "I am experiencing menstruation,” "Menstruation is disappearing,” and "No menstruation for over a year.” Women who selected one of the first two options were classified as premenopausal, whereas those who selected the third option were classified as postmenopausal.

### Collection of data for the remaining study variables

Further information regarding the data collection for the remaining study variables is provided in the Supplementary Material.

## Statistical analysis

Stratified analyses were performed after categorizing the participants into two subgroups (premenopausal and postmenopausal women) based on their menopausal status. Continuous variables were presented as mean (standard deviation [SD]) or median (interquartile range), as appropriate, whereas categorical variables were expressed as numbers (proportions). Differences in the characteristics between analyzed participants and those excluded due to missing or clinically improbable data were assessed using the Student’s *t*-test or chi-square test.

We first performed analyses only on multiparous women (excluding nulliparous women), considering the potential differences in the characteristics between nulliparous and multiparous women due to the varying medical or socioeconomic backgrounds or personal preferences affecting childbirth decisions. Participants with a parity of 1 were set as the reference category for premenopausal and postmenopausal women. The linear relationship between parity and CKD prevalence was examined using the Cochran–Armitage test. Multiple logistic regression models were used to explore the relationship between parity and CKD prevalence. Model 1 was adjusted for age. Model 2 was additionally adjusted for height, physical activity, marital status, smoking status, alcohol consumption, participant’s birth weight, highest educational level, family history of type 2 diabetes mellitus, family history of hypertension, family history of glomerulonephritis, breastfeeding experience, oral contraceptive use, hormone replacement therapy use, thyroid dysfunction [21], endometriosis, mental disease, menstrual cycle, age at menarche (<15 years or ≥15 years), age at last delivery (<35 years or ≥35 years), sleeping time, nap time, year of study participation, Prefecture (Miyagi or Iwate), and the number of relocations after the GEJE. We included menopausal age (age at menopause <40 years or ≥40 years) when postmenopausal women were analyzed in Model 2. In addition to the Model 2 variables, Model 3 was adjusted for γ-GTP (<50 or ≥50 IU) based on a previous study [22, 23] and for the estimated 24-h sodium chloride (NaCl) and potassium (K) intakes, abnormal levels of which were associated with CKD [24, 25]. The intakes were calculated based on previously reported methods [26, 27]. In addition to the Model 3 variables, Model 4 was adjusted for the HDP and GDM clinical history. Based on previous studies that showed that parity was associated with obesity [10, 28], Model 5 was adjusted for BMI at age 20 years, per 1-SD increase, in addition to the Model 4 variables. Model 6 was adjusted for the current BMI per 1-SD increase in addition to the Model 4 variables. Furthermore, the linear relationship between parity and CKD prevalence was evaluated in each model.

In addition, the relationship between parity and CKD prevalence was investigated in all premenopausal and postmenopausal women (nulliparous and multiparous women). Women with a parity of 1 were set as the reference category. Model 1 was adjusted for age. Model 2 was adjusted for the covariates previously mentioned, except for breastfeeding experience and the age at last delivery (<35 or ≥35 years). In addition to the Model 2 covariates, Model 3 was adjusted for γ-GTP (<50 or ≥50 IU) [22, 23] and the estimated 24-h NaCl and K intakes [26, 27]. Model 4 was adjusted for BMI at age 20 per 1-SD increase, in addition to the Model 3 covariates. Furthermore, in addition to the Model 3 covariates, Model 5 was adjusted for the current BMI per 1-SD increase.

The general linear model was used to confirm the absence of a strong multicollinearity. Multiple imputations using a Markov chain Monte Carlo simulation were used to compensate for missing data in several covariates. The dependent variable (CKD) and all the covariates were used to create the imputation model. Notably, each dataset was separately analyzed after generating 20 datasets using multiple imputations, and the 20 results were combined using Rubin's rule [29].

Participant characteristics were analyzed using the gtsummary package of R version 4.1.1 [30]. Other statistical analyses were performed using SAS version 9.4 (SAS Institute Inc., Cary, North Carolina, USA).

## Results

### Inclusion and exclusion criteria

**Fig 1:**
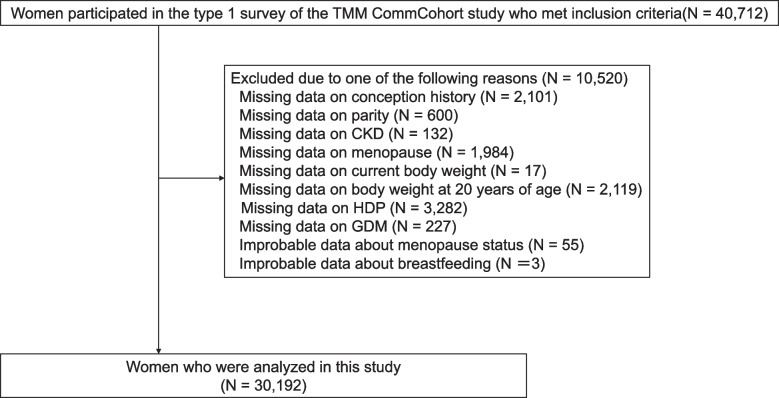
Study’s flow chart

Figure 1 shows a flowchart depicting our study’s screening and selection of participants. Among the 40,712 women who participated in the type 1 survey of the TMM CommCohort Study and met the inclusion criteria, the following were excluded due to missing data about conception history (*N*=2,101), parity (*N*=600), CKD (*N*=132), menopause (*N*=1,984), current body weight (BW) (*N*=17), BW at 20 years of age (*N*=2,119), clinical history of HDP (*N*=3,282), clinical history of GDM (*N*=227), or improbable data about menopausal status (*N*=55) and breastfeeding (*N*=3). Ultimately, the study included 30,192 women.

### Characteristics of premenopausal participants

Table 1 presents the characteristics of the premenopausal participants stratified based on parity. The average age of this study’s premenopausal participants was 41.2 years, and 4.9% of them had CKD. As parity increased, higher age, hypertension prevalence, proportions of hormone replacement therapy, clinical history of HDP, and residence in Iwate Prefecture were observed. The proportion of women with current obesity was the highest among women with parity ≥4. The proportions of unmarried and divorced women were higher among women with parities of 0 and 1. The number of women with high levels of education increased with decreasing parity.

**Table 1:** Characteristics of premenopausal participants

**Variables**		**Parity**				
	**All** **(*N*=6,605)**	**0 (*N*=1,599)**^**a**^	**1 (*N*=1,110)**^**a**^	**2 (*N*=2,448)**^**a**^	**3 (*N*=1,186)**^**a**^	**≥4 (*N*=262)**^**a**^
**Chronic kidney disease, N (%)**	321 (4.9)	77 (4.8)	53 (4.8)	119 (4.9)	52 (4.4)	20 (7.6)
**Hypertension, N (%)**	801 (12.1)	163 (10.2)	116 (10.5)	294 (12.0)	182 (15.3)	46 (17.6)
**Type 2 diabetes, N (%)**	138 (2.1)	36 (2.3)	21 (1.9)	39 (1.6)	35 (3.0)	7 (2.7)
**Age, years**	41.2 (7.4)	38.1 (8.3)	39.9 (6.9)	42.4 (6.6)	43.7 (6.7)	44.3 (6.7)
**Category of age, N (%)**						
20–29.9 years	426 (6.4)	298 (18.6)	71 (6.4)	47 (1.9)	8 (0.7)	2 (0.8)
30–39.9 years	2,255 (34.1)	560 (35.0)	454 (40.9)	810 (33.1)	357 (30.1)	74 (28.2)
40–49.9 years	2,933 (44.4)	618 (38.6)	483 (43.5)	1,172 (47.9)	549 (46.3)	111 (42.4)
50–59.9 years	991 (15.0)	123 (7.7)	102 (9.2)	419 (17.1)	272 (22.9)	75 (28.6)
**Height, cm**	157.3 (5.3)	157.4 (5.4)	157.4 (5.4)	157.1 (5.2)	157.2 (5.2)	156.6 (4.9)
**Body weight, kg**	55.2 (10.3)	55.7 (12.1)	54.9 (10.2)	54.6 (9.3)	55.5 (9.4)	56.6 (9.8)
**BMI, kg/m**^**2**^	22.3 (4.0)	22.5 (4.7)	22.2 (4.0)	22.1 (3.7)	22.4 (3.6)	23.1 (4.0)
**Category of BMI, N (%)**						
Underweight (<18.5 kg/m^2^)	842 (12.7)	262 (16.4)	154 (13.9)	296 (12.1)	108 (9.1)	22 (8.4)
Normal range (18.5–24.9 kg/m^2^)	4,413 (66.8)	975 (61.0)	741 (66.8)	1,714 (70.0)	819 (69.1)	164 (62.6)
Obese (≥25.0 kg/m^2^)	1,350 (20.4)	362 (22.6)	215 (19.4)	438 (17.9)	259 (21.8)	76 (29.0)
**Body weight at age 20 years, kg**	52.0 (8.0)	53.4 (9.7)	51.8 (8.2)	51.2 (6.9)	51.6 (7.3)	52.4 (7.3)
**Body weight gain after 20 years, kg**	3.2 (8.0)	2.4 (8.4)	3.1 (8.5)	3.4 (7.4)	3.8 (8.2)	4.2 (8.2)
**Waist circumference, cm**	78.4 (9.8)	78.0 (11.2)	78.5 (9.9)	78.2 (9.0)	78.9 (9.1)	80.4 (10.2)
**Waist circumference ≥90 cm, N (%)**	781 (11.9)	217 (13.7)	128 (11.6)	251 (10.3)	140 (11.8)	45 (17.2)
**Physical activity level, METS, median (IQR)**	26.8 (20.9–34.3)	26.8 (21.8–34.2)	25.9 (20.4–33.7)	26.6 (20.8–33.9)	27.3 (20.8–36.2)	27.9 (21.1–37.0)
**Smoking status, N (%)**						
Never smoker	4,507 (68.2)	1,145 (71.6)	703 (63.3)	1,665 (68.0)	822 (69.3)	172 (65.6)
Ever smoker	1,153 (17.5)	213 (13.3)	230 (20.7)	464 (19.0)	198 (16.7)	48 (18.3)
Current smoker	925 (14.0)	237 (14.8)	177 (15.9)	309 (12.6)	160 (13.5)	42 (16.0)
Missing	20 (0.3)	4 (0.3)	0 (0.0)	10 (0.4)	6 (0.5)	0 (0.0)
**Alcohol consumption, N (%)**						
Never drinker	3,298 (49.9)	836 (52.3)	561 (50.5)	1,207 (49.3)	564 (47.6)	130 (49.6)
Ever drinker	188 (2.8)	35 (2.2)	79 (7.1)	54 (2.2)	17 (1.4)	3 (1.1)
Current drinker	3,098 (46.9)	721 (45.1)	466 (42.0)	1,182 (48.3)	601 (50.7)	128 (48.9)
Missing	21 (0.3)	7 (0.4)	4 (0.4)	5 (0.2)	4 (0.3)	1 (0.4)
**Γ-GTP ≥50 IU, N (%)**	346 (5.2)	90 (5.6)	69 (6.2)	112 (4.6)	66 (5.6)	9 (3.4)
**Estimated 1-day NaCl intake**	9.2 (2.3)	8.9 (2.3)	9.1 (2.3)	9.4 (2.3)	9.5 (2.3)	9.7 (2.4)
**Estimated 1-day K intake**	1,998.2 (494.5)	1,911.2 (498.2)	1,979.4 (480.2)	2,022.5 (487.4)	2,069.0 (503.6)	2,061.9 (477.8)
**Own birth weight, N (%)**						
<2,500 g	567 (8.6)	177 (11.1)	101 (9.1)	201 (8.2)	68 (5.7)	20 (7.6)
2,500–3,499 g	4,746 (71.9)	1,094 (68.4)	802 (72.3)	1,783 (72.8)	872 (73.5)	195 (74.4)
≥3,500 g	743 (11.2)	204 (12.8)	132 (11.9)	271 (11.1)	114 (9.6)	22 (8.4)
Unknown	434 (6.6)	83 (5.2)	64 (5.8)	160 (6.5)	108 (9.1)	19 (7.3)
Missing	115 (1.7)	41 (2.6)	11 (1.0)	33 (1.3)	24 (2.0)	6 (2.3)
**History of thyroid disease, N (%)**						
Yes	206 (3.1)	45 (2.8)	30 (2.7)	84 (3.4)	38 (3.2)	9 (3.4)
No	6,216 (94.1)	1,378 (86.2)	1,078 (97.1)	2,361 (96.4)	1,146 (96.6)	253 (96.6)
Missing	183 (2.8)	176 (11.0)	2 (0.2)	3 (0.1)	2 (0.2)	0 (0.0)
**History of endometriosis, N (%)**						
Yes	326 (4.9)	87 (5.4)	73 (6.6)	117 (4.8)	41 (3.5)	8 (3.1)
No	6,102 (92.4)	1,336 (83.6)	1,037 (93.4)	2,330 (95.2)	1,145 (96.5)	254 (96.9)
Missing	177 (2.7)	176 (11.0)	0 (0.0)	1 (0.0)	0 (0.0)	0 (0.0)
**Mental disease, N (%)**						
Yes	389 (5.9)	158 (9.9)	69 (6.2)	116 (4.7)	28 (2.4)	18 (6.9)
No	6,036 (91.4)	1,268 (79.3)	1,039 (93.6)	2,329 (95.1)	1,156 (97.5)	244 (93.1)
Missing	180 (2.7)	173 (10.8)	2 (0.2)	3 (0.1)	2 (0.2)	0 (0.0)
**Breastfeeding experience, N (%)**						
Yes	4,747 (71.9)	0 (0.0)	1,018 (91.7)	2,340 (95.6)	1,133 (95.5)	256 (97.7)
No	1,794 (27.2)	1,546 (96.7)	86 (7.7)	106 (4.3)	50 (4.2)	6 (2.3)
Missing	64 (1.0)	53 (3.3)	6 (0.5)	2 (0.1)	3 (0.3)	0 (0.0)
**Experience with oral contraceptives, N (%)**						
Yes	286 (4.3)	59 (3.7)	58 (5.2)	97 (4.0)	53 (4.5)	19 (7.3)
No	6,174 (93.5)	1,440 (90.1)	1,040 (93.7)	2,328 (95.1)	1,126 (94.9)	240 (91.6)
Missing	145 (2.2)	100 (6.3)	12 (1.1)	23 (0.9)	7 (0.6)	3 (1.1)
**Experience with hormone replacement therapy, N (%)**						
Yes	164 (2.5)	26 (1.6)	19 (1.7)	65 (2.7)	41 (3.5)	13 (5.0)
No	6,272 (95.0)	1,467 (91.7)	1,073 (96.7)	2,350 (96.0)	1,137 (95.9)	245 (93.5)
Missing	169 (2.6)	106 (6.6)	18 (1.6)	33 (1.3)	8 (0.7)	4 (1.5)
**Age <15 years at menarche, N (%)**						
<15 years	6,157 (93.2)	1,470 (91.9)	1,022 (92.1)	2,319 (94.7)	1,104 (93.1)	242 (92.4)
≥15 years	407 (6.2)	115 (7.2)	78 (7.0)	122 (5.0)	76 (6.4)	16 (6.1)
Missing	41 (0.6)	14 (0.9)	10 (0.9)	7 (0.3)	6 (0.5)	4 (1.5)
**Age ≥35 years at last delivery, N (%)**						
<35 years	3,722 (56.4)	0 (0.0)	804 (72.4)	1,914 (78.2)	863 (72.8)	141 (53.8)
≥35 years	1,173 (17.8)	0 (0.0)	268 (24.1)	480 (19.6)	305 (25.7)	120 (45.8)
Missing	1,710 (25.9)	-	38 (3.4)	54 (2.2)	18 (1.5)	1 (0.4)
**Menstrual cycle, N (%)**						
Regular	5,156 (78.1)	1,222 (76.4)	846 (76.2)	1,965 (80.3)	927 (78.2)	196 (74.8)
Irregular	1,394 (21.1)	366 (22.9)	253 (22.8)	463 (18.9)	247 (20.8)	65 (24.8)
Missing	55 (0.8)	11 (0.7)	11 (1.0)	20 (0.8)	12 (1.0)	1 (0.4)
**History of HDP, N (%)**	290 (4.4)	0 (0.0)	48 (4.3)	138 (5.6)	79 (6.7)	25 (9.5)
**History of GDM, N (%)**	51 (0.8)	0 (0.0)	12 (1.1)	19 (0.8)	17 (1.4)	3 (1.1)
**Family history of glomerulonephritis, N (%)**						
Yes	52 (0.8)	12 (0.8)	7 (0.6)	28 (1.1)	4 (0.3)	1 (0.4)
No	6,367 (96.4)	1,407 (88.0)	1,102 (99.3)	2,417 (98.7)	1,180 (99.5)	261 (99.6)
Missing	186 (2.8)	180 (11.3)	1 (0.1)	3 (0.1)	2 (0.2)	0 (0.0)
**Family history of hypertension, N (%)**						
Yes	2,927 (44.3)	749 (46.8)	470 (42.3)	1,076 (44.0)	512 (43.2)	120 (45.8)
No	3,569 (54.0)	748 (46.8)	638 (57.5)	1,369 (55.9)	672 (56.7)	142 (54.2)
Missing	109 (1.7)	102 (6.4)	2 (0.2)	3 (0.1)	2 (0.2)	0 (0.0)
**Family history of type 2 diabetes, N (%)**						
Yes	894 (13.5)	249 (15.6)	151 (13.6)	318 (13.0)	141 (11.9)	35 (13.4)
No	5,541 (83.9)	1,188 (74.3)	956 (86.1)	2,127 (86.9)	1,043 (87.9)	227 (86.6)
Missing	170 (2.6)	162 (10.1)	3 (0.3)	3 (0.1)	2 (0.2)	0 (0.0)
**Marital status, N (%)**						
Married	5,015 (75.9)	502 (31.4)	941 (84.8)	2,237 (91.4)	1,098 (92.6)	237 (90.5)
Unmarried	1,054 (16.0)	1,017 (63.6)	23 (2.1)	8 (0.3)	4 (0.3)	2 (0.8)
Divorced	400 (6.1)	60 (3.8)	129 (11.6)	148 (6.0)	47 (4.0)	16 (6.1)
Widowed	120 (1.8)	10 (0.6)	15 (1.4)	55 (2.2)	33 (2.8)	7 (2.7)
Missing	16 (0.2)	10 (0.6)	2 (0.2)	0 (0.0)	4 (0.3)	0 (0.0)
**Highest level of education, N (%)**						
Low	212 (3.2)	46 (2.9)	36 (3.2)	68 (2.8)	46 (3.9)	16 (6.1)
Medium	4,823 (73.0)	1,098 (68.7)	793 (71.4)	1,811 (74.0)	909 (76.6)	212 (80.9)
High	1,527 (23.1)	440 (27.5)	277 (25.0)	553 (22.6)	223 (18.8)	34 (13.0)
Missing	43 (0.7)	15 (0.9)	4 (0.4)	16 (0.7)	8 (0.7)	0 (0.0)
**Frequency of breakfast, N (%)**						
Everyday	5,270 (79.8)	1,067 (66.7)	922 (83.1)	2,086 (85.2)	998 (84.1)	197 (75.2)
Skipping	1,311 (19.8)	530 (33.1)	185 (16.7)	357 (14.6)	178 (15.0)	61 (23.3)
Missing	24 (0.4)	2 (0.1)	3 (0.3)	5 (0.2)	10 (0.8)	4 (1.5)
**Sleeping time, N (%)**						
<7 h	4,994 (75.6)	1,119 (70.0)	802 (72.3)	1,897 (77.5)	959 (80.9)	217 (82.8)
≥7 and ≤8 h	1,210 (18.3)	331 (20.7)	229 (20.6)	443 (18.1)	178 (15.0)	29 (11.1)
≥8 h	395 (6.0)	147 (9.2)	78 (7.0)	107 (4.4)	47 (4.0)	16 (6.1)
Missing	6 (0.1)	2 (0.1)	1 (0.1)	1 (0.0)	2 (0.2)	0 (0.0)
**Nap time, N (%)**						
Not taking a nap	4,492 (68.0)	1,092 (68.3)	753 (67.8)	1,684 (68.8)	806 (68.0)	157 (59.9)
Nap time is < 1 h/day	1,431 (21.7)	317 (19.8)	217 (19.5)	542 (22.1)	281 (23.7)	74 (28.2)
Nap time is ≥ 1 h/day	656 (9.9)	185 (11.6)	135 (12.2)	211 (8.6)	94 (7.9)	31 (11.8)
Missing	26 (0.4)	5 (0.3)	5 (0.5)	11 (0.4)	5 (0.4)	0 (0.0)
**Number of relocations after the GEJE, N (%)**						
0	4,623 (70.0)	1,087 (68.0)	684 (61.6)	1,768 (72.2)	890 (75.0)	194 (74.0)
1	866 (13.1)	224 (14.0)	204 (18.4)	299 (12.2)	112 (9.4)	27 (10.3)
2	508 (7.7)	139 (8.7)	99 (8.9)	171 (7.0)	84 (7.1)	15 (5.7)
3	314 (4.8)	73 (4.6)	64 (5.8)	109 (4.5)	55 (4.6)	13 (5.0)
≥4	214 (3.2)	56 (3.5)	43 (3.9)	73 (3.0)	35 (3.0)	7 (2.7)
Missing	80 (1.2)	20 (1.3)	16 (1.4)	28 (1.1)	10 (0.8)	6 (2.3)
**Year, N (%)**						
2013	1,051 (15.9)	360 (22.5)	148 (13.3)	318 (13.0)	182 (15.3)	43 (16.4)
2014	2,788 (42.2)	663 (41.5)	495 (44.6)	1,020 (41.7)	490 (41.3)	120 (45.8)
2015	2,766 (41.9)	576 (36.0)	467 (42.1)	1,110 (45.3)	514 (43.3)	99 (37.8)
**Prefecture, N (%)**						
Miyagi	4,138 (62.6)	1,065 (66.6)	736 (66.3)	1,536 (62.7)	673 (56.7)	128 (48.9)
Iwate	2,467 (37.4)	534 (33.4)	374 (33.7)	912 (37.3)	513 (43.3)	134 (51.1)

^a^Continuous and categorical variables are shown as means (standard deviations) and numbers (percentages), respectively

*Abbreviations:* *BMI* body mass index, *HDP* hypertensive disorders of pregnancy, *GDM* gestational diabetes mellitus, *γ-GTP* γ-glutamyl transpeptidase, *GEJE* Great East Japan Earthquake

### Characteristics of postmenopausal participants

Table 2 depicts the characteristics of the postmenopausal women stratified based on parity. The average age of postmenopausal women was 63.9 years, and 10.9% of them had CKD. The mean value of current BMI and the proportion of current obesity increased with parity. However, the proportion of those with a family history of glomerulonephritis, hypertension, or T2DM decreased with parity. The proportions of unmarried and divorced women were higher in nulliparous women and women with a parity of 1, whereas that of women with a high level of education was the highest in the nulliparous group.

**Table 2:** Characteristics of postmenopausal participants

		**Parity**				
**Variables**	**All** **(*N* = 23,587)**^**a**^	**0 (*N*=1,648)**^**a**^	**1 (*N*=2,083)**^**a**^	**2 (*N*=11,467)**^**a**^	**3 (*N*=7,169)**^**a**^	**≥4 (*N*=1,220)**^**a**^
**Chronic kidney disease, N (%)**	2,573 (10.9)	148 (9.0)	238 (11.4)	1,236 (10.8)	794 (11.1)	157 (12.9)
**Hypertension, N (%)**	9,778 (41.5)	577 (35.0)	803 (38.6)	4,832 (42.1)	3,039 (42.4)	527 (43.2)
**Type 2 diabetes, N (%)**	1,876 (8.0)	108 (6.6)	151 (7.3)	880 (7.7)	610 (8.5)	127 (10.4)
**Age, years**	63.9 (6.2)	61.6 (7.0)	63.3 (7.4)	64.4 (5.9)	63.9 (5.9)	63.7 (6.2)
**Category of age, N (%)**						
20–29.9 years	12 (0.1)	0 (0.0)	8 (0.4)	2 (0.0)	2 (0.0)	0 (0.0)
30–39.9 years	59 (0.3)	6 (0.4)	14 (0.7)	24 (0.2)	12 (0.2)	3 (0.2)
40–49.9 years	283 (1.2)	74 (4.5)	55 (2.6)	87 (0.8)	52 (0.7)	15 (1.2)
50–59.9 years	4,798 (20.3)	506 (30.7)	433 (20.8)	2,074 (18.1)	1,507 (21.0)	278 (22.8)
60–69.9 years	13,591 (57.6)	829 (50.3)	1,119 (53.7)	6,746 (58.8)	4,223 (58.9)	674 (55.2)
≥70 years	4,844 (20.5)	233 (14.1)	454 (21.8)	2,534 (22.1)	1,373 (19.2)	250 (20.5)
**Height, cm**	152.1 (5.6)	153.3 (5.9)	152.2 (5.8)	152.0 (5.5)	152.1 (5.5)	151.5 (5.7)
**Body weight, kg**	53.4 (8.6)	52.5 (9.4)	52.8 (9.1)	52.8 (8.4)	54.3 (8.5)	55.1 (8.8)
**BMI, kg/m**^**2**^	23.1 (3.6)	22.3 (3.8)	22.8 (3.8)	22.9 (3.5)	23.5 (3.5)	24.0 (3.6)
**Category of BMI, N (%)**						
Underweight (<18.5 kg/m^2^)	1,791 (7.6)	234 (14.2)	209 (10.0)	906 (7.9)	400 (5.6)	42 (3.4)
Normal range (18.5–24.9 kg/m^2^)	15,825 (67.1)	1,070 (64.9)	1,408 (67.6)	7,881 (68.7)	4,704 (65.6)	762 (62.5)
Obese (≥25.0 kg/m^2^)	5,971 (25.3)	344 (20.9)	466 (22.4)	2,680 (23.4)	2,065 (28.8)	416 (34.1)
**Body weight at age 20 years, kg**	51.0 (7.4)	51.2 (8.6)	50.3 (7.9)	50.6 (6.9)	51.6 (7.6)	51.5 (7.7)
**Weight gain after 20 years, kg**	2.4 (9.1)	1.3 (9.7)	2.5 (9.7)	2.2 (8.7)	2.7 (9.3)	3.6 (9.9)
**Waist circumference, cm**	82.0 (9.1)	80.4 (10.0)	81.2 (9.5)	81.6 (8.9)	83.0 (9.0)	83.9 (9.2)
**Waist circumference ≥90 cm, N (%)**	4,163 (17.7)	263 (16.0)	337 (16.2)	1,832 (16.0)	1,438 (20.1)	293 (24.0)
**Physical activity level, METS, median (IQR)**	28.2 (21.8-37.7)	26.9 (21.2-34.0)	26.8 (20.5-35.1)	28.0 (21.7-37.0)	29.0 (22.2-39.7)	30.9 (23.0-42.2)
**Smoking status, N (%)**						
Never smoker	20,595 (87.3)	1,303 (79.1)	1,724 (82.8)	10,117 (88.2)	6,395 (89.2)	1,056 (86.6)
Ever smoker	1,459 (6.2)	191 (11.6)	194 (9.3)	665 (5.8)	338 (4.7)	71 (5.8)
Current smoker	964 (4.1)	129 (7.8)	122 (5.9)	402 (3.5)	258 (3.6)	53 (4.3)
Missing	569 (2.4)	25 (1.5)	43 (2.1)	283 (2.5)	178 (2.5)	40 (3.3)
**Alcohol consumption, N (%)**						
Never drinking	15,645 (66.3)	1,006 (61.0)	1,418 (68.1)	7,692 (67.1)	4,697 (65.5)	832 (68.2)
Ever drinking	337 (1.4)	29 (1.8)	45 (2.2)	152 (1.3)	90 (1.3)	21 (1.7)
Current drinker	7,372 (31.3)	604 (36.7)	604 (29.0)	3,508 (30.6)	2,306 (32.2)	350 (28.7)
Missing	233 (1.0)	9 (0.5)	16 (0.8)	115 (1.0)	76 (1.1)	17 (1.4)
**Γ-GTP ≥50 IU, N (%)**	1,832 (7.8)	143 (8.7)	149 (7.2)	870 (7.6)	559 (7.8)	111 (9.1)
**Estimated 1-day NaCl intake**	9.8 (2.2)	9.5 (2.2)	9.6 (2.2)	9.7 (2.2)	9.9 (2.3)	9.9 (2.2)
**Estimated 1-day K intake**	2,169.8 (483.0)	2,162.3 (486.8)	2,138.0 (486.5)	2,162.8 (476.8)	2,188.4 (492.7)	2,190.4 (467.5)
**Own birth weight, N (%)**						
<2,500 g	2,259 (9.6)	181 (11.0)	220 (10.6)	1,083 (9.4)	668 (9.3)	107 (8.8)
2,500–3,499 g	7,965 (33.8)	671 (40.7)	705 (33.8)	3,688 (32.2)	2,477 (34.6)	424 (34.8)
≥3,500 g	528 (2.2)	73 (4.4)	48 (2.3)	227 (2.0)	150 (2.1)	30 (2.5)
Unknown	11,609 (49.2)	661 (40.1)	996 (47.8)	5,859 (51.1)	3,509 (48.9)	584 (47.9)
Missing	1,226 (5.2)	62 (3.8)	114 (5.5)	610 (5.3)	365 (5.1)	75 (6.1)
**History of thyroid disease, N (%)**						
Yes	1,349 (5.7)	103 (6.2)	103 (4.9)	674 (5.9)	405 (5.6)	64 (5.2)
No	22,059 (93.5)	1,408 (85.4)	1,976 (94.9)	10,771 (93.9)	6,750 (94.2)	1,154 (94.6)
Missing	179 (0.8)	137 (8.3)	4 (0.2)	22 (0.2)	14 (0.2)	2 (0.2)
**History of endometriosis, N (%)**						
Yes	1,093 (4.6)	157 (9.5)	154 (7.4)	509 (4.4)	245 (3.4)	28 (2.3)
No	22,359 (94.8)	1,356 (82.3)	1,929 (92.6)	10,958 (95.6)	6,924 (96.6)	1,192 (97.7)
Missing	135 (0.6)	135 (8.2)	0 (0.0)	0 (0.0)	0 (0.0)	0 (0.0)
**Mental disease, N (%)**						
Yes	686 (2.9)	72 (4.4)	83 (4.0)	319 (2.8)	172 (2.4)	40 (3.3)
No	22,704 (96.3)	1,434 (87.0)	1,994 (95.7)	11,120 (97.0)	6,978 (97.3)	1,178 (96.6)
Missing	197 (0.8)	142 (8.6)	6 (0.3)	28 (0.2)	19 (0.3)	2 (0.2)
**Breastfeeding experience, N (%)**						
Yes	19,172 (81.3)	0 (0.0)	1,592 (76.4)	9,847 (85.9)	6,570 (91.6)	1,163 (95.3)
No	4,237 (18.0)	1,592 (96.6)	458 (22.0)	1,569 (13.7)	566 (7.9)	52 (4.3)
Missing	178 (0.8)	56 (3.4)	33 (1.6)	51 (0.4)	33 (0.5)	5 (0.4)
**Experience with oral contraceptives, N (%)**						
Yes	628 (2.7)	31 (1.9)	53 (2.5)	267 (2.3)	238 (3.3)	39 (3.2)
No	22,221 (94.2)	1,374 (83.4)	1,963 (94.2)	10,955 (95.5)	6,778 (94.5)	1,151 (94.3)
Missing	738 (3.1)	243 (14.7)	67 (3.2)	245 (2.1)	153 (2.1)	30 (2.5)
**Experience with hormone replacement therapy, N (%)**						
Yes	1,807 (7.7)	127 (7.7)	178 (8.5)	903 (7.9)	500 (7.0)	99 (8.1)
No	21,180 (89.8)	1,336 (81.1)	1,854 (89.0)	10,359 (90.3)	6,538 (91.2)	1,093 (89.6)
Missing	600 (2.5)	185 (11.2)	51 (2.4)	205 (1.8)	131 (1.8)	28 (2.3)
**Age <15 years at menarche, N (%)**						
<15 years	18,211 (77.2)	1,386 (84.1)	1,604 (77.0)	8,731 (76.1)	5,551 (77.4)	939 (77.0)
≥15 years	5,138 (21.8)	249 (15.1)	454 (21.8)	2,621 (22.9)	1,550 (21.6)	264 (21.6)
Missing	238 (1.0)	13 (0.8)	25 (1.2)	115 (1.0)	68 (0.9)	17 (1.4)
**Age ≥35 years at last delivery, N (%)**						
<35 years	19,426 (82.4)	0 (0.0)	1,721 (82.6)	10,645 (92.8)	6,210 (86.6)	850 (69.7)
≥35 years	2,093 (8.9)	0 (0.0)	275 (13.2)	627 (5.5)	844 (11.8)	347 (28.4)
Missing	2,068 (8.8)	-	87 (4.2)	195 (1.7)	115 (1.6)	23 (1.9)
**Menstrual cycle, N (%)**						
Regular	18,188 (77.1)	1,232 (74.8)	1,510 (72.5)	8,849 (77.2)	5,658 (78.9)	939 (77.0)
Irregular	3,928 (16.7)	347 (21.1)	415 (19.9)	1,938 (16.9)	1,049 (14.6)	179 (14.7)
Missing	1,471 (6.2)	69 (4.2)	158 (7.6)	680 (5.9)	462 (6.4)	102 (8.4)
**History of HDP, N (%)**	1,021 (4.3)	0 (0.0)	110 (5.3)	545 (4.8)	315 (4.4)	51 (4.2)
**History of GDM, N (%)**	33 (0.1)	0 (0.0)	5 (0.2)	17 (0.1)	9 (0.1)	2 (0.2)
**Family history of glomerulonephritis, N (%)**						
Yes	93 (0.4)	14 (0.8)	15 (0.7)	43 (0.4)	20 (0.3)	1 (0.1)
No	23,312 (98.8)	1,486 (90.2)	2,061 (98.9)	11,412 (99.5)	7,135 (99.5)	1,218 (99.8)
Missing	182 (0.8)	148 (9.0)	7 (0.3)	12 (0.1)	14 (0.2)	1 (0.1)
**Family history of hypertension, N (%)**						
Yes	8,555 (36.3)	777 (47.1)	782 (37.5)	4,172 (36.4)	2,453 (34.2)	371 (30.4)
No	14,968 (63.5)	824 (50.0)	1,300 (62.4)	7,290 (63.6)	4,707 (65.7)	847 (69.4)
Missing	64 (0.3)	47 (2.9)	1 (0.0)	5 (0.0)	9 (0.1)	2 (0.2)
**Family history of type 2 diabetes, N (%)**						
Yes	2,476 (10.5)	251 (15.2)	226 (10.8)	1,193 (10.4)	698 (9.7)	108 (8.9)
No	20,944 (88.8)	1,276 (77.4)	1,851 (88.9)	10,252 (89.4)	6,455 (90.0)	1,110 (91.0)
Missing	167 (0.7)	121 (7.3)	6 (0.3)	22 (0.2)	16 (0.2)	2 (0.2)
**Marital status, N (%)**						
Married	18,530 (78.6)	726 (44.1)	1,555 (74.7)	9,342 (81.5)	5,927 (82.7)	980 (80.3)
Unmarried	854 (3.6)	697 (42.3)	27 (1.3)	71 (0.6)	47 (0.7)	12 (1.0)
Divorced	975 (4.1)	68 (4.1)	201 (9.6)	444 (3.9)	215 (3.0)	47 (3.9)
Widowed	3,072 (13.0)	144 (8.7)	289 (13.9)	1,534 (13.4)	940 (13.1)	165 (13.5)
Missing	156 (0.7)	13 (0.8)	11 (0.5)	76 (0.7)	40 (0.6)	16 (1.3)
**Highest level of education, N (%)**						
Low	5,299 (22.5)	192 (11.7)	458 (22.0)	2,483 (21.7)	1,741 (24.3)	425 (34.8)
Medium	15,384 (65.2)	1,090 (66.1)	1,346 (64.6)	7,697 (67.1)	4,600 (64.2)	651 (53.4)
High	2,644 (11.2)	352 (21.4)	256 (12.3)	1,167 (10.2)	742 (10.4)	127 (10.4)
Missing	260 (1.1)	14 (0.8)	23 (1.1)	120 (1.0)	86 (1.2)	17 (1.4)
**Frequency of breakfast, N (%)**						
Everyday	21,751 (92.2)	1,461 (88.7)	1,892 (90.8)	10,630 (92.7)	6,649 (92.7)	1,119 (91.7)
Skipping	1,391 (5.9)	169 (10.3)	156 (7.5)	615 (5.4)	379 (5.3)	72 (5.9)
Missing	445 (1.9)	18 (1.1)	35 (1.7)	222 (1.9)	141 (2.0)	29 (2.4)
**Sleeping time, N (%)**						
<7 h	17,449 (74.0)	1,201 (72.9)	1,573 (75.5)	8,526 (74.4)	5,287 (73.7)	862 (70.7)
≥7 and <=8 h	4,723 (20.0)	338 (20.5)	399 (19.2)	2,273 (19.8)	1,457 (20.3)	256 (21.0)
≥8 h	1,392 (5.9)	107 (6.5)	110 (5.3)	657 (5.7)	418 (5.8)	100 (8.2)
Missing	23 (0.1)	2 (0.1)	1 (0.0)	11 (0.1)	7 (0.1)	2 (0.2)
**Nap time, N (%)**						
Not taking a nap	14,309 (60.7)	1,113 (67.5)	1,302 (62.5)	7,103 (61.9)	4,109 (57.3)	682 (55.9)
Nap time is < 1 h/day	7,647 (32.4)	397 (24.1)	621 (29.8)	3,596 (31.4)	2,592 (36.2)	441 (36.1)
Nap time is ≥ 1 h/day	1,521 (6.4)	131 (7.9)	151 (7.2)	723 (6.3)	432 (6.0)	84 (6.9)
Missing	110 (0.5)	7 (0.4)	9 (0.4)	45 (0.4)	36 (0.5)	13 (1.1)
**Number of relocations after the GEJE, N (%)**						
0	18,185 (77.1)	1,236 (75.0)	1,571 (75.4)	8,887 (77.5)	5,557 (77.5)	934 (76.6)
1	1,367 (5.8)	130 (7.9)	118 (5.7)	669 (5.8)	380 (5.3)	70 (5.7)
2	995 (4.2)	90 (5.5)	102 (4.9)	462 (4.0)	293 (4.1)	48 (3.9)
3	961 (4.1)	70 (4.2)	101 (4.8)	442 (3.9)	313 (4.4)	35 (2.9)
≥4	513 (2.2)	42 (2.5)	59 (2.8)	254 (2.2)	134 (1.9)	24 (2.0)
Missing	1,566 (6.6)	80 (4.9)	132 (6.3)	753 (6.6)	492 (6.9)	109 (8.9)
**Year, N (%)**						
2013	4,010 (17.0)	351 (21.3)	371 (17.8)	1,811 (15.8)	1,194 (16.7)	283 (23.2)
2014	10,817 (45.9)	776 (47.1)	968 (46.5)	5,351 (46.7)	3,228 (45.0)	494 (40.5)
2015	8,760 (37.1)	521 (31.6)	744 (35.7)	4,305 (37.5)	2,747 (38.3)	443 (36.3)
**Prefecture, N (%)**						
Miyagi	12,499 (53.0)	885 (53.7)	1,054 (50.6)	6,267 (54.7)	3,789 (52.9)	504 (41.3)
Iwate	11,088 (47.0)	763 (46.3)	1,029 (49.4)	5,200 (45.3)	3,380 (47.1)	716 (58.7)
**Menopause age, N (%)**						
Premature menopause (age at menopause <40 years)	909 (3.9)	107 (6.5)	139 (6.7)	418 (3.6)	209 (2.9)	36 (3.0)
Postmenopause (age at menopause ≥40 years)	22,134 (93.8)	1,509 (91.6)	1,887 (90.6)	10,808 (94.3)	6,780 (94.6)	1,150 (94.3)
Missing	544 (2.3)	32 (1.9)	57 (2.7)	241 (2.1)	180 (2.5)	34 (2.8)
**Reasons for menopause, N (%)**						
Natural menopause	19,083 (80.9)	1,228 (74.5)	1,545 (74.2)	9,328 (81.3)	5,974 (83.3)	1,008 (82.6)
Menopause due to surgery of the uterus and/or ovary	3,439 (14.6)	305 (18.5)	393 (18.9)	1,698 (14.8)	900 (12.6)	143 (11.7)
Other reasons	762 (3.2)	66 (4.0)	116 (5.6)	330 (2.9)	204 (2.8)	46 (3.8)
Missing	303 (1.3)	49 (3.0)	29 (1.4)	111 (1.0)	91 (1.3)	23 (1.9)

^a^Continuous and categorical variables are shown as mean (standard deviation) and number (percentage), respectively

*Abbreviations:* *BMI* body mass index, *HDP* hypertensive disorders of pregnancy, *GDM* gestational diabetes mellitus, *γ-GTP* γ-glutamyl transpeptidase, *GEJE* Great East Japan Earthquak

### Relationship between parity and CKD in premenopausal multiparous women

**Fig 2:**
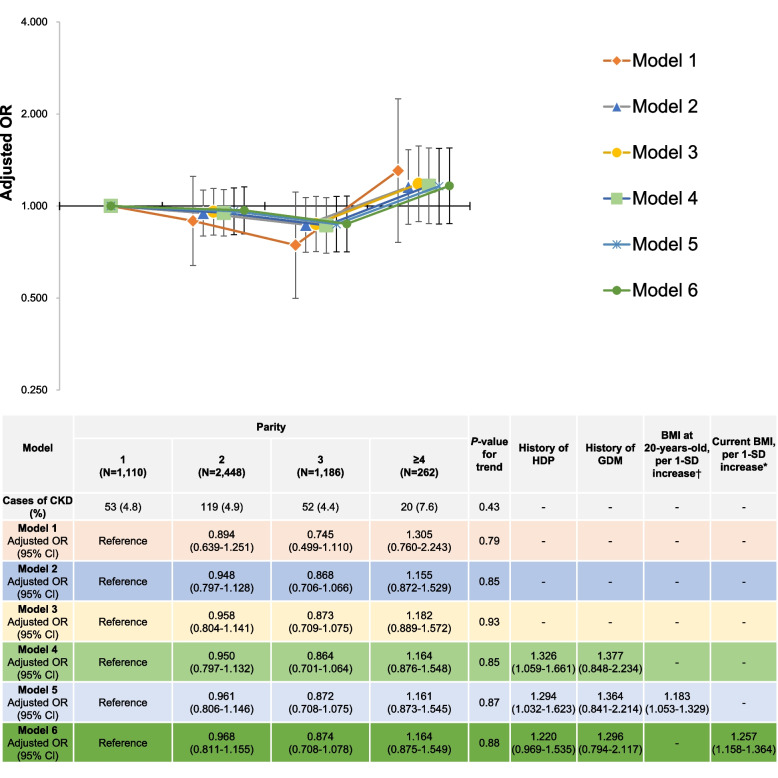
Relationship between parity and CKD in premenopausal multiparous women. †1-SD value was 2.8 kg/m2 for BMI at 20-years-old. * 1-SD value was 3.8 kg/m2 for current BMI. Model 1: Adjusting for age. Model 2: Model 1 variables in addition to height, physical activity, marital status, smoking status, alcohol consumption, own birth weight, highest educational level, family history of type 2 diabetes mellitus, family history of hypertension, family history of glomerulonephritis, breastfeeding experience, oral contraceptive use, hormone replacement therapy use, thyroid dysfunction, endometriosis, mental disease, menstrual cycle, age at menarche (<15 years or ≥15 years), age at last delivery (<35 years or ≥35 years), sleeping time, nap time, year of study participation, prefecture (Miyagi or Iwate), and number of relocations after the GEJE. Model 3: Model 2 variables, γ-GTP (<50 or ≥50 IU/l), and estimated 24 h NaCl and K intakes. Model 4: Model 3 variables, a clinical history of HDP and a clinical history of GDM. Model 5: Model 4 variables and BMI at 20-years-old, as per 1-SD increase. Model 6: Model 4 and current BMI as per 1-SD increase. Abbreviations: CKD chronic kidney disease, BMI body mass index, CI confidence interval, HDP hypertensive disorders of pregnancy, GDM gestational diabetes mellitus, GEJE Great East Japan Earthquake, γ-GTP γ-Glutamyl transpeptidase, OR odds ratio, SD standard deviation, NaCl sodium chloride, K, potassium

Figure 2 shows the relationship between parity and CKD prevalence in premenopausal multiparous women. Women with a parity of 3 had lower odds for CKD prevalence; however, the results were not significant. No significant graded linear relationship was observed between parity and CKD prevalence in Models 1, 2, and 3 (*P*-value for trend: 0.79, 0.85, and 0.93 in Models 1, 2, and 3, respectively) or Model 4 (*P*-value for trend: 0.85). In addition, no significant linear relationship between parity and CKD prevalence was observed in Models 5 and 6 (*P*-value for trend: 0.87 and 0.88, respectively). In Models 5 and 6, BMI at 20 years old and current BMI were associated with CKD prevalence (adjusted odds ratio [OR] per 1-SD increase in BMI at 20 years and current BMI: 1.183 [95% confidence interval [CI]: 1.053-1.329] and 1.257 [95% CI:1.158-1.364]), respectively. Model 4 showed that a history of HDP was associated with CKD prevalence (adjusted OR: 1.326 [95% CI: 1.059–1.661]). A history of HDP remained a risk factor for CKD prevalence after adjusting for BMI at age 20 years (adjusted OR: 1.294 [95% CI: 1.032–1.623] in Model 5; however, this was attenuated after adjusting for current BMI (adjusted OR: 1.220 [95% CI: 0.969–1.535]) in Model 6.

### Relationship between parity and CKD in postmenopausal multiparous women

**Fig 3:**
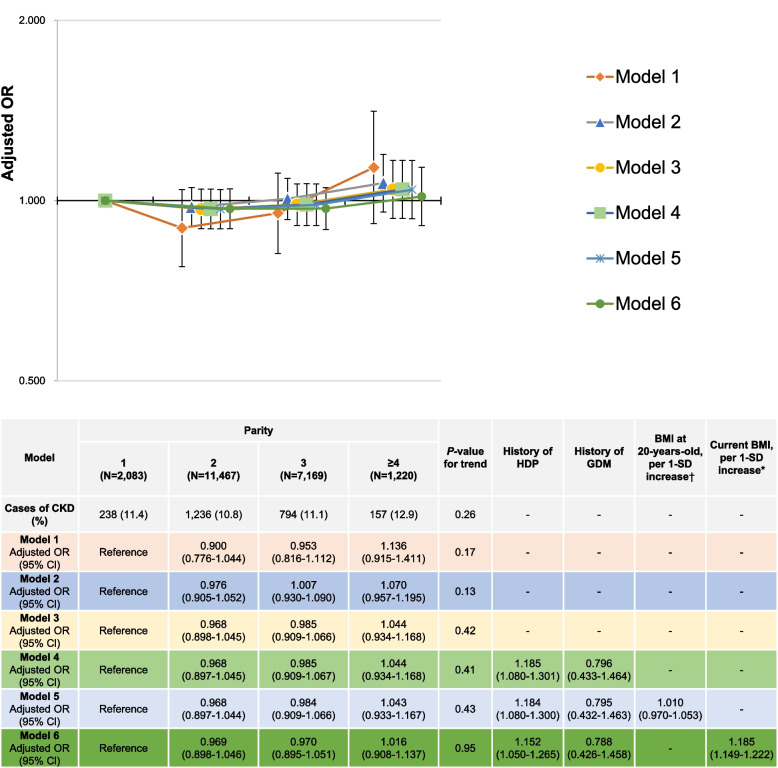
Relationship between parity and CKD prevalence in postmenopausal multiparous women.†1-SD value was 3.1 kg/m2 for BMI at 20-years-old. *1-SD value was 3.6 kg/m2 for current BMI. Model 1: Adjusting for age. Model 2: Model 1 variables in addition to height, physical activity, marital status, smoking status, alcohol consumption, own birth weight, highest educational level, family history of hypertension, family history of type 2 diabetes mellitus, family history of glomerulonephritis, breastfeeding experience, oral contraceptive use, hormone replacement therapy use, thyroid dysfunction, endometriosis, mental disease, menstrual cycle, age at menarche (<15 years or ≥15 years), age at last delivery (<35 years or ≥35 years), menopause age (<40 years or ≥40 years), sleeping time, nap time, year of study participation, prefecture (Miyagi or Iwate), and number of relocations after the GEJE. Model 3: Model 2 variables, γ-GTP (<50 or ≥50 IU/l), and estimated 24 h NaCl and K intakes. Model 4: Model 3 variables and a clinical history of HDP and a clinical history of GDM. Model 5: Model 4 variables and BMI at 20-years-old as per 1-SD increase. Model 6: Model 4 variables and current BMI as per 1-SD increase. Abbreviations: CKD chronic kidney disease, BMI body mass index, CI, confidence interval, HDP, hypertensive disorders of pregnancy, GDM gestational diabetes mellitus, GEJE Great East Japan Earthquake, γ-GTP, γ-glutamyl transpeptidase, OR odds ratio, SD, standard deviation, NaCl sodium chloride, K, potassium

Figure 3 shows the relationship between parity and CKD prevalence in postmenopausal multiparous women. Women with a parity of ≥4 had higher odds for CKD prevalence, but the results were not significant. No significant graded linear relationship was observed between parity and CKD prevalence in Models 1, 2, and 3 (*P*-value for trend: 0.17, 0.13, and 0.42 in Models 1, 2, and 3, respectively) or Model 4 (*P*-value for trend: 0.41). Models 5 and 6 showed no significant linear relationship between parity and CKD prevalence (*P*-value for trend: 0.43 and 0.95, respectively). In Model 5, BMI at age 20 years was not associated with CKD prevalence (adjusted OR per 1-SD increase in BMI at age 20 years: 1.010 [95% CI: 0.970-1.053]). In Model 6, current BMI was associated with CKD prevalence (adjusted OR per 1-SD increase in current BMI: 1.185 [95% CI: 1.149-1.222]). Furthermore, a history of HDP was associated with CKD prevalence (adjusted OR: 1.185 [95% CI: 1.080–1.301]) in Model 4. However, a history of HDP remained a risk factor for CKD even after adjusting for BMI at age 20 years (adjusted OR: 1.184 [95% CI: 1.080–1.300] in Model 5, and current BMI (adjusted OR: 1.152 [95% CI: 1.050–1.265]) in Model 6.

### Relationship between parity and CKD in all premenopausal women

The results of the relationship between parity and CKD prevalence in all premenopausal women are shown in Supplementary Figure 1 and Material.

### Relationship between parity and CKD in all postmenopausal women

The results of the relationship between parity and CKD prevalence in all postmenopausal women are presented in Supplementary Figure 2 and Material.

### Combined analysis for the investigation of the interaction between a clinical history of HDP and current BMI in premenopausal multiparous women

The results are presented in Supplementary Figure 3 and Material.

### Combined analysis for the investigation of the interaction between a clinical history of HDP and current BMI in postmenopausal multiparous women

The results are shown in Supplementary Figure 4 and Material.

## Discussion

To the best of our knowledge, this is the first study to examine the relationship between parity and CKD prevalence in Japan. No significant association was observed between parity and CKD prevalence in premenopausal and postmenopausal women. Therefore, high parity does not necessarily increase the risk of CKD. However, our study contradicted the findings of Sun et al. [7], who found parity to be associated with a higher CKD prevalence in middle-aged and older Chinese women. Differences in ethnicity, lifestyle, the proportion of the number of parity (most women in Sun’s study were women with a parity of 1), and study design could have led to these different results.

A clinical history of HDP was associated with the risk of CKD, except when adjusting for current BMI in premenopausal women. These findings are consistent with those of Oishi et al.[12] and Barrett et al.[31], who showed that HDP increased the risk of CKD. Therefore, it is crucial to consider the potential mechanisms underlying the relationship between a clinical history of HDP and CKD prevalence. Pre-eclampsia, a subtype of HDP, leads to glomerular endotheliosis, resulting in glomerular dysfunction and subsequent microalbuminuria [32, 33]. Primary renal injury due to podocyte loss is also associated with pre-eclampsia that persists after pregnancy, resulting in CKD [34, 35]. Therefore, establishing evidence to reduce the risk of preeclampsia through interventions such as low-dose oral aspirin use, which could attenuate kidney dysfunction, is necessary in Japan [36, 37].

Furthermore, the adjusted OR for CKD in participants with a clinical history of HDP tended to be higher than that for those with a current BMI < 25.0 kg/m^2^ in this study. Weight loss reduces albuminuria and slows the decline in eGFR [38]; therefore, maintaining an appropriate BW would help reduce the risk of CKD, especially in women with a clinical history of HDP.

Our study’s strengths include its large sample size and the various covariates considered, including medical history, lifestyle habits, and social factors. However, this study has some limitations. The study did not examine the risk of CKD over time and used a single result of eGFR and urine ACR, which could lead to misclassification of CKD. Owing to the small population of women with a higher stage of CKD, the association of parity with individual CKD stages could not be estimated. In addition, this study relied on self-reported information, and this may have introduced recall bias and influenced the results’ accuracy. However, based on previous studies, the number of children recorded in self-reported questionnaires was almost identical to that in medical records; therefore, this limitation did not significantly influence this study’s results [39]. Furthermore, this study did not collect information on multiple pregnancies, which may be relevant to the association between parity and CKD. HDP was not defined until 1982 in Japan [40]; therefore, women who gave birth before 1982 were not diagnosed with HDP, resulting in its underestimation. The absence of stillbirth data limited the consideration of its association with HDP. Another limitation is the absence of preconception evaluation for creatinine/eGFR and albuminuria levels to rule out underlying CKD as a factor in HDP/pre-eclampsia development, potentially reversing the causality.

Despite these limitations, this study provides valuable preliminary evidence on the relationship between parity and CKD prevalence considering the limited global research on this topic. The clinical history of HDP also highlights this study’s importance. As parity is associated with hypertension and this association is attenuated after adjusting for current BMI [41], it is notable that the influence of pregnancy differs based on blood pressure and kidney function. Therefore, further prospective studies with larger sample sizes and longitudinal follow-ups are needed to confirm these findings and investigate the potential mechanisms underlying this association.

## Conclusions

Parity is not significantly associated with CKD prevalence. A clinical history of HDP is a risk factor for CKD in both premenopausal and postmenopausal women. Current BMI is also associated with an increased risk of CKD in premenopausal and postmenopausal women. Therefore, continuous surveillance and preventive measures against CKD should be provided for women with a clinical history of HDP, and all women should be encouraged to maintain an appropriate body weight.

### Supplementary Information

Supplementary Material 1.

## Data Availability

The data and materials that support this study’s findings are available upon reasonable request and procedures, with permission from dist@megabank.tohoku.ac.jp. Please contact Noriyuki Iwama (Email address: noriyuki.iwama.a3@tohoku.ac.jp).

## Abbreviations

CKD: chronic kidney disease
BMI: body mass index
HDP: hypertensive disorders of pregnancy
ESRD: end-stage renal disease
TMM CommCohort Study: Tohoku Medical Megabank Community-based Cohort Study
GEJE: Great East Japan Earthquake
