# Supplementary material for: Relationship between parity and the prevalence of chronic kidney disease in Japan considering hypertensive disorders of pregnancy and body mass index
Source: BMC Nephrol. 2024 May 17;25:166. doi: 10.1186/s12882-024-03604-z (PMC11100170; doi:10.1186/s12882-024-03604-z)
Supplement: Supplementary file 1 — Supplementary Material 1. [file 12882_2024_3604_MOESM1_ESM.pdf]

SUPPLEMENTARY MATERIAL

Relationship between parity and the prevalence of chronic kidney disease in Japan considering hypertensive disorders of pregnancy and body mass index

Hongxin Wang[a], Noriyuki Iwama[a,b,c], Keiichi Yuwaki[d], You Nakamichi[d], Hirotaka Hamada[a], Hasumi Tomita[a], Kazuma Tagami[a], Rie Kudo[a], Natsumi Kumagai[a], Hirohito Metoki[c,e], Naoki Nakaya[c], Atsushi Hozawa[c], Shinichi Kuriyama[f,g,h], Nobuo Yaegashi[a,b,c,h], and Masatoshi Saito[a,b,i]

[a] Department of Obstetrics and Gynecology, Tohoku University Graduate School of Medicine, 1-1, Seiryomachi, Sendai 980-8574, Miyagi, Japan

[b] Women's Health Care Medical Science, Tohoku University Graduate School of Medicine, 1-1, Seiryomachi, Sendai 980-8574, Miyagi, Japan

[c] Tohoku Medical Megabank Organization, Tohoku University, 2-1, Seiryomachi, Sendai 980-8573, Miyagi, Japan

[d] Underwriting and Medical Department, The Dai-ichi Life Insurance Company, Limited, Koto-ku, Tokyo, Japan

[e] Division of Public Health, Hygiene and Epidemiology, Tohoku Medical Pharmaceutical University, 1-15-1 Fukumuro, Sendai 983-8536, Miyagi, Japan

[f] Division of Molecular Epidemiology, Tohoku University Graduate School of Medicine, 1-1, Seiryomachi, Sendai 980-8574, Miyagi, Japan

[g] International Research Institute of Disaster Science, Tohoku University, 468-1, Aramaki, Sendai 980-8572, Miyagi, Japan

[h] Environment and Genome Research Center, Tohoku University Graduate School of Medicine, 2-1, Seiryomachi, Sendai 980-8575, Miyagi, Japan Sendai, Miyagi, Japan

[I] Department of Maternal and Fetal Therapeutics, Tohoku University Graduate School of Medicine, 1-1, Seiryomachi, Sendai 980-8574, Miyagi, Japan

Corresponding Author

Noriyuki Iwama, MD, PhD

Department of Obstetrics and Gynecology, Tohoku University Hospital

1-1, Seiryomachi, Sendai 980-8574, Miyagi, Japan

Phone: +81-22-717-7251; Fax: +81-22-717-7258
Email: noriyuki.iwama.a3@ tohoku.ac.jp

1. Data collection for the remaining study variables

Data from municipal health checkups were acquired to collect information on age, height, current body weight (BW), current waist circumference (WC), and blood and urine test results. The BW at age 20 years was acquired from the self-reported questionnaire, and the corresponding body mass index (BMI) was calculated as weight (kg) divided by the square of the height (m). Following the Western Pacific Region of World Health Organization criteria for Japanese individuals, the BMI at age 20 was grouped as underweight (<18.5 kg/m^2), normal (≥18.5 and <25.0 kg/m^2), or obese (≥25.0 kg/m^2)[1]. Current BW and height were used to calculate current BMI, and participants were categorized as underweight, normal weight, or obese. The weight gain was calculated as the difference between the current BW and the BW at age 20. The γ-GTP level was classified into <50 and ≥50 IU based on a previous study[1,2]. The estimated 24-h sodium chloride (NaCl) and potassium (K) intakes were calculated using previously reported methods[3,4].

We obtained information from the self-reported questionnaire on marital status, smoking, alcohol drinking, own birth weight, highest education level, family history of glomerulonephritis, family history of hypertension, family history of type 2 diabetes mellitus (T2DM), breastfeeding experience, oral contraceptive use, hormone replacement therapy use, hyperthyroidism, hypothyroidism, endometriosis, depression before the GEJE, depression after the GEJE, bipolar disorder, schizophrenia, menstrual cycle, age at menarche, age at last delivery, average sleeping time/day, nap time, year of participation in the TMM CommCohort Study, prefecture (Miyagi or Iwate), and number of relocations after the GEJE.

Marital status was categorized into three groups: “married or widowed,” “unmarried,” and “divorced.” Smoking status was divided into three groups: never smoker, ever smoker, and current smoker. Women who reported smoking < 100 cigarettes in their lifetime were categorized as never smoker, those who had smoked > 100 cigarettes in their lifetime and were currently smoking were categorized as current smoker, and those who smoked > 100 cigarettes during their lifetime but were currently not smoking were categorized as ever smoker. Alcohol drinking status was classified as non-drinker, ever drinker, and current

drinker based on whether they rarely drank or could not drink, having quit alcohol now, and currently drinking, respectively. The participants' birth weight was categorized as <2,500, ≥2,500, <3,500, ≥3,500 g, and unknown. The highest educational level was categorized as low (elementary school or junior high school), medium (high school or vocational school), or high (college, technical college, university, or graduate school), according to a previous study.[5] Participants who chose other answers were categorized into the missing data group. A family history of glomerulonephritis, hypertension, or T2DM was defined as a history of glomerulonephritis, hypertension, or T2DM, respectively, in the father, mother, brother, or sister. Thyroid dysfunction was classified as hyperthyroidism or hypothyroidism. Mental diseases were classified as depression before the GEJE, depression after the GEJE, bipolar disorder, and schizophrenia.

The metabolic equivalents (METs) assigned to physical activity were calculated based on the information in the questionnaire, which quantifies physical activity based on a previous study[5]. Delayed menarche was classified as the age at menarche ≥15 years, according to the Japan Society of Obstetrics and Gynecology[6]. Advanced maternal age at the last delivery was defined as

the age at the last delivery of ≥35 years. Breakfast skipping was defined as a frequency of breakfast less than once/month, once to three times/month, once to twice/week, three to four times/week, or five to six times/week.

The average sleeping time/day was categorized into <7, ≥7 and <8, and ≥8 h based on a previous study[7]. We categorized nap time into not taking a nap, nap <1 h/day, and nap ≥1 h/day. Premature menopause was defined as menopausal age <40 years in postmenopausal women. The reasons for menopause were grouped into natural menopause, surgical menopause due to the removal of the uterus and/or ovary, and menopause due to other reasons. The years of study participation were 2013, 2014, and 2015. The number of relocations after the GEJE was classified as 0, 1, 2, 3, and ≥4.

2. Relationship between parity and CKD prevalence in all premenopausal women (nulliparous and multiparous women)

The relationship between parity and CKD prevalence in all premenopausal women is shown in Supplementary Figure 1. Models 1, 2, and 3 showed no significant linear relationship between parity and hypertension prevalence (P-values for trend: 0.19, 0.40, and 0.48 in models 1, 2, and 3,

respectively). In Model 4, no significant association was observed (P-value for trend: 0.51). The BMI at age 20 was associated with CKD prevalence (adjusted odds ratio (OR) per 1-standard deviation (SD) increase in BMI at age 20:1.208 [95% confidence interval (CI): 1.089–1.339]). No significant linear relationship between parity and CKD prevalence was observed (P-value for trend: 0.51) in Model 5. Current BMI was significantly associated with CKD prevalence (adjusted OR per 1-SD increase in current BMI: 1.216 [95% CI: 1.136–1.302]).

3. Relationship between parity and CKD prevalence in all postmenopausal women (nulliparous and multiparous women)

Supplementary Figure 2 depicts the relationship between parity and CKD prevalence in Models 1-5 for all postmenopausal women. Nulliparous women had a lower risk of CKD than women with a parity of 1. However, after excluding women with a clinical history of hypertension, or type 2 diabetes mellitus, the nulliparous women no longer had a lower risk of CKD (data not shown). The higher prevalence of CKD complications among multiparous women may have influenced our results.

BMI at age 20 was not associated with increased CKD prevalence (adjusted OR per 1-SD increase in BMI at age 20:1.015 [95% CI: 0.973–1.058]). Current BMI was significantly associated with an increased risk of CKD (adjusted OR per 1-SD increase in current BMI: 1.198 [95% CI: 1.162–1.235]).

4. Combined analysis for the investigation of the interaction between a clinical history of HDP and current BMI in multiparous women

A combined analysis was conducted to investigate the interaction between the clinical history of HDP and current BMI in multiparous women. Current BMI was divided into two categories: non-obese (BMI <25.0 kg/m^2) and obese (BMI ≥25.0 kg/m^2). In this analysis, non-obese women with no clinical history of HDP were used as the reference category. 5 models are defined. Models 1, 2, and 3 were adjusted for the same covariates used in the principal analysis. Model 4 was adjusted for a history of GDM, in addition to those of Model 3. Model 5 was adjusted for BMI at 20 years of age following the 1-SD increase, in addition to Model 4 covariates.

The results for premenopausal multiparous and postmenopausal multiparous women are shown in Supplementary Figures 3 and 4, respectively.

The interaction between a clinical history of HDP and current BMI (non-obese or obese) was not statistically significant, except in Model 1.

In premenopausal and postmenopausal women, women with obesity had a higher risk of CKD than those without obesity. Premenopausal obese women with a clinical history of HDP had a higher risk of developing CKD than those without HDP. Among the postmenopausal women, those with a clinical history of HDP had a higher risk of developing CKD than those without HDP, regardless of their obesity.

5. Differences in characteristics between participants who were analyzed and those who were excluded due to missing or clinically improbable data

Supplementary Table 1 shows the differences in characteristics between analyzed participants and those excluded due to missing or clinically improbable data. Participants who were excluded were older and had a higher prevalence of CKD than those who were analyzed. They also had higher proportions of participants with obesity, body weight gain, prevalence of hypertension, lower levels of education, lower proportions of nulliparous

women, and underweight women. Most of the participants excluded were from the year 2013, and there were more residents of Miyagi prefecture.

6. Association between parity and urine albumin-to-creatinine ratio ≥ 300 mg/gCre in postmenopausal women

Supplementary Table 2 shows the association between parity and urine albumin-to-creatinine ratio (ACR) ≥300 mg/gCre in postmenopausal multiparous women (women with parity ≥1). Supplementary Table 3 shows the association between parity and urine ACR ≥ 300 mg/gCre in all postmenopausal women (including nulliparous women). The models were adjusted for the same covariates used in the analysis to investigate the association between parity and CKD prevalence. No significant association between parity and urine ACR ≥300 mg/gCre was observed. A history of HDP and current BMI were associated with a high risk of urine ACR ≥300 mg/gCre. Meanwhile, analysis relative to the association between parity and urine ACR≥ 300 mg/gCre was not feasible in premenopausal women owing to the small number of women with urine ACR ≥ 300 mg/gCre (n=25 women) in this population.

7. References

1. Itabashi F, Hirata T, Kogure M, Narita A, Tsuchiya N, Nakamura T, et al. Combined associations of liver enzymes and obesity with diabetes mellitus prevalence: the Tohoku medical megabank community-based cohort study. J Epidemiol. 2022;32:221-7.
2. Ikai E, Noborizaka Y, Tsuritani I, Honda R, Ishizaki M, Yamada Y. Serum gamma-glutamyl transpeptidase levels and hypertension in non-drinkers: a possible role of fatty liver in the pathogenesis of obesity related hypertension. Obes Res. 1993;1:469-74.
3. Tanaka T, Okamura T, Miura K, Kadowaki T, Ueshima H, Nakagawa H, et al. A simple method to estimate populational 24-h urinary sodium and potassium excretion using a casual urine specimen. J Hum Hypertens. 2002;16:97-103.
4. Tasevska N, Runswick SA, Bingham SA. Urinary potassium is as reliable as urinary nitrogen for use as a recovery biomarker in dietary studies of free living individuals. J Nutr. 2006;136:1334-40.
5. Nakaya N, Xie T, Scheerder B, Tsuchiya N, Narita A, Nakamura T, et al. Spousal similarities in cardiometabolic risk factors: A cross-sectional

comparison between Dutch and Japanese data from two large biobank studies. Atherosclerosis. 2021;334:85-92.

6. Shozu M, Ishikawa H, Horikawa R, Sakakibara H, Izumi SI, Ohba T, et al. Nomenclature of primary amenorrhea: A proposal document of the Japan Society of Obstetrics and Gynecology committee for the redefinition of primary amenorrhea. J Obstet Gynaecol Res. 2017;43:1738-42.

7. Heianza Y, Kato K, Fujihara K, Tanaka S, Kodama S, Hanyu O, et al. Role of sleep duration as a risk factor for Type 2 diabetes among adults of different ages in Japan: the Niigata Wellness Study. Diabet Med. 2014;31:1363-7.

Supplementary Figure Legends

Supplementary Fig 1. Relationship between parity and CKD prevalence in all premenopausal women

†1-SD value was 3.0 kg/m2 for BMI at 20-years-old. * 1-SD value was 4.0 kg/m2 for current BMI.

Model 1: Adjusting for age.

Model 2: Model 1 variables in addition to height, physical activity, marital status, smoking status, alcohol consumption, own birth weight, highest educational level, family history of type 2 diabetes mellitus, family history of hypertension, family history of glomerulonephritis, oral contraceptive use, hormone replacement therapy use, thyroid dysfunction, endometriosis, mental disease, menstrual cycle, age at menarche (<15 years or ≥15 years), sleeping time, nap time, year of study participation, prefecture (Miyagi or Iwate), and number of relocations after the GEJE.

Model 3: Model 2 variables, γ-GTP (<50 or ≥50 IU/l), and estimated 24 h NaCl and K intakes.

Model 4: Model 3 variables and BMI at 20 years old, as per 1-SD increase.

Model 5: Model 3 variables and current BMI, as per 1-SD increase.

Abbreviations: CKD, chronic kidney disease; BMI, body mass index; CI, confidence interval; GEJE, Great East Japan Earthquake; γ-GTP, γ-glutamyl transpeptidase; OR, odds ratio; SD, standard deviation; NA, not applicable; NaCl, sodium chloride; K, potassium.

Supplementary Fig 2. Relationship between parity and CKD prevalence in all postmenopausal women.

†1-SD value was 3.1 kg/m2 for BMI at 20-years-old. *1-SD value was 3.6 kg/m2 for current BMI.

Model 1: Adjusting for age.

Model 2: Model 1 variables in addition to height, physical activity, marital status, smoking status, alcohol consumption, own birth weight, highest educational level, family history of hypertension, family history of type 2 diabetes mellitus, family history of glomerulonephritis, oral contraceptive use, hormone replacement therapy use, thyroid dysfunction, endometriosis, mental disease, menstrual cycle, age at menarche (<15 years or ≥15 years), menopause age (<40 years or ≥40 years), sleeping time, nap time, year of study participation, Prefecture (Miyagi or Iwate), and number of relocations after the GEJE.

Model 3: Model 2 variables, γ-GTP (<50 IU/l or ≥50 IU/l), and estimated 24 h NaCl and K intakes.

Model 4: Model 3 variables and BMI at 20 years old, as per 1-SD increase.

Model 5: Model 3 variables and current BMI, as per 1-SD increase.

Abbreviations: CKD, chronic kidney disease; BMI, body mass index; CI, confidence interval; GEJE, Great East Japan Earthquake; γ-GTP, γ-glutamyl transpeptidase; OR, odds ratio; SD, standard deviation; NaCl, sodium chloride; K, potassium.

Supplementary Fig 3. Combined analysis for the investigation of the interaction between a clinical history of HDP and obesity in premenopausal multiparous women.

†1-SD value was 2.8 kg/m^2 for BMI at 20-years-old.

Model 1: Adjusting for age.

Model 2: Model 1 variables in addition to height, physical activity, marital status, smoking status, alcohol consumption, own birth weight, highest educational level, family history of type 2 diabetes mellitus, family history of hypertension, family history of glomerulonephritis, breastfeeding experience, oral contraceptive use,

hormone replacement therapy use, thyroid dysfunction, endometriosis, mental disease, menstrual cycle, age at menarche (<15 years or ≥15 years), age at last delivery (<35 years or ≥35 years), sleeping time, nap time, year of study participation, prefecture (Miyagi or Iwate), and number of relocations after the GEJE.

Model 3: Model 2 variables, γ-GTP (<50 IU/l or ≥50 IU/l), and estimated 24 h NaCl and K intakes.

Model 4: Model 3 variables and a history of GDM.

Model 5: Model 4 variables and BMI at 20 years old, as per 1-SD increase.

Abbreviations: CKD, chronic kidney disease; BMI, body mass index; CI, confidence interval; HDP, hypertensive disorders of pregnancy; GDM, gestational diabetes mellitus; GEJE, Great East Japan Earthquake; γ-GTP, γ-glutamyl transpeptidase; OR, odds ratio; SD, standard deviation; NaCl, sodium chloride; K, potassium.

Supplementary Fig 4. Combined analysis for the investigation of the interaction between a clinical history of HDP and obesity in postmenopausal multiparous women.

†1-SD value was 3.1 kg/m^2 for BMI at 20-years-old.

Model 1: Adjusting for age.

Model 2: Model 1 variables in addition to height, physical activity, marital status, smoking status, alcohol consumption, own birth weight, highest educational level, family history of type 2 diabetes mellitus, family history of hypertension, family history of glomerulonephritis, breastfeeding experience, oral contraceptive use, hormone replacement therapy use, thyroid dysfunction, endometriosis, mental disease, menstrual cycle, age at menarche (<15 years or ≥15 years), age at last delivery (<35 years or ≥35 years), menopause age (<40 years or ≥40 years), sleeping time, nap time, year of study participation, prefecture (Miyagi or Iwate), and number of relocations after the GEJE.

Model 3: Model 2 variables, γ-GTP (<50 IU/l or ≥50 IU/l), and estimated 24 h NaCl and K intakes.

Model 4: Model 3 variables and a history of GDM.

Model 5: Model 4 variables and BMI at 20 years old, as per 1-SD increase.

Abbreviations: CKD, chronic kidney disease; BMI, body mass index; CI, confidence interval; HDP, hypertensive disorders of pregnancy; GDM, gestational diabetes mellitus; GEJE, Great East Japan Earthquake; γ-GTP, γ-glutamyl

transpeptidase; OR, odds ratio; SD, standard deviation; NaCl, sodium chloride; K, potassium.

Supplementary Table 1. Differences in the characteristics between participants who were analyzed and those who were excluded due to missing or clinically improbable data

**Variables**	**Study participants (N=30,192)[1]**	**Excluded participants (N=10,520)[1]**	***P*-value[2]**
**Parity, N (%)**			<0.001
0	3,247 (10.8)	262 (3.3)	
1	3,193 (10.6)	896 (11.4)	
2	13,915 (46.1)	3,891 (49.6)	
3	8,355 (27.7)	2,356 (30.0)	
≥4	1,482 (4.9)	446 (5.7)	
**Age, years**	59.0 (11.4)	60.6 (11.7)	<0.001
**Category of age, N (%)**			<0.001
20-29.9 years	438 (1.5)	154 (1.5)	
30-39.9 years	2,314 (7.7)	733 (7.0)	
40-49.9 years	3,216 (10.7)	949 (9.0)	
50-59.9 years	5,789 (19.2)	1,634 (15.5)	
60-69.9 years	13,591 (45.0)	4,446 (42.3)	

Supplementary Table 1. Differences in the characteristics between participants who were analyzed and those who were excluded due to missing or clinically improbable data

Variables	Study participants (N=30,192)[1]	Excluded participants (N=10,520)[1]	*P*-value[2]
≥70 years	4,844 (16.0)	2,604 (24.8)	
**Height, cm**	153.2 (5.9)	152.3 (6.1)	<0.001
**Body weight, kg**	53.8 (9.0)	54.6 (9.4)	<0.001
**BMI, kg/m²**	22.9 (3.7)	23.5 (3.9)	<0.001
**Category of BMI, N (%)**			<0.001
Underweight (<18.5 kg/m²)	2,633 (8.7)	719 (6.9)	
Normal range (18.5-24.9 kg/m²)	20,238 (67.0)	6,517 (62.1)	
Obese (≥25.0 kg/m²)	7,321 (24.2)	3,258 (31.0)	
**Body weight at age 20 years, kg**	51.2 (7.5)	51.1 (7.3)	0.599
**BMI at age 20 years, kg/m²**	21.8 (3.1)	21.9 (3.2)	0.113
**Weight gain after 20 years, kg**	2.6 (8.9)	3.3 (9.2)	<0.001
**Waist circumference ≥90 cm, N (%)**	4,944 (16.4)	2,167 (20.7)	<0.001
**Waist circumference, cm**	81.2 (9.4)	82.5 (9.8)	<0.001

Supplementary Table 1. Differences in the characteristics between participants who were analyzed and those who were excluded due to missing or clinically improbable data

**Variables**	**Study participants (N=30,192)**[1]	**Excluded participants (N=10,520)**[1]	***P*-value**[2]
**Chronic kidney disease, N (%)**			<0.001
No	27,298 (90.4)	9,167 (87.1)	
Yes	2,894 (9.6)	1,203 (11.4)	
Missing	0 (0.0)	150 (1.4)	
**Estimated GFR**	105 (22)	99 (22)	<0.001
**Albumin-creatinine ratio, mg/gCr**	19 (92)	23 (141)	<0.001
**Hypertension, N (%)**	10,579 (35.0)	3,884 (36.9)	<0.001
**Type 2 diabetes, N (%)**	2,014 (6.7)	731 (7.0)	0.342
**SBP, mmHg**	124 (18)	126 (18)	<0.001
**DBP, mmHg**	74 (10)	74 (11)	<0.001
**History of glomerulonephritis, N (%)**			<0.001
No	29,663 (98.2)	4,775 (45.4)	

Supplementary Table 1. Differences in the characteristics between participants who were analyzed and those who were excluded due to missing or clinically improbable data

Variables	Study participants (N=30,192)[1]	Excluded participants (N=10,520)[1]	*P*-value[2]
Yes	166 (0.5)	27 (0.3)	
Missing	363 (1.2)	5,718 (54.4)	
**History of kidney dialysis, N (%)**			<0.001
No	29,813 (98.7)	4,824 (45.9)	
Yes	14 (0.0)	2 (0.0)	
missing	365 (1.2)	5,694 (54.1)	
**Physical activity level, METS, median (IQR)**	27.8 (21.5-36.9)	27.1 (20.3-36.6)	0.007
**Smoking status, N (%)**			<0.001
Never smoker	25,102 (83.1)	7,196 (68.4)	
Ever smoker	2,612 (8.7)	726 (6.9)	
Current smoker	1,889 (6.3)	563 (5.4)	
Missing	589 (2.0)	2,035 (19.3)	

Supplementary Table 1. Differences in the characteristics between participants who were analyzed and those who were excluded due to missing or clinically improbable data

Variables	Study participants (N=30,192)[1]	Excluded participants (N=10,520)[1]	*P*-value[2]
**Alcohol consumption, N (%)**			<0.001
Never drinker	18,943 (62.7)	5,817 (55.3)	
Ever drinker	525 (1.7)	138 (1.3)	
Current drinker	10,470 (34.7)	2,803 (26.6)	
Missing	254 (0.8)	1,762 (16.7)	
**Γ-GTP ≥50 IU, N (%)**	2,178 (7.2)	851 (8.1)	0.003
**Estimated 1-day NaCl intake**	9.7 (2.3)	9.7 (2.3)	0.305
**Estimated 1-day K intake**	2,132.2 (490.7)	2,074.5 (483.9)	<0.001
**Own birth weight, N (%)**			<0.001
<2,500 g	2,826 (9.4)	767 (7.3)	
2,500-3,499 g	12,711 (42.1)	3,033 (28.8)	
≥3,500 g	1,271 (4.2)	293 (2.8)	
Unknown	12,043 (39.9)	3,510 (33.4)	

Supplementary Table 1. Differences in the characteristics between participants who were analyzed and those who were excluded due to missing or clinically improbable data

**Variables**	**Study participants (N=30,192)[1]**	**Excluded participants (N=10,520)[1]**	***P*-value[2]**
Missing	1,341 (4.4)	2,917 (27.7)	
**History of thyroid disease, N (%)**			<0.001
Yes	1,555 (5.2)	388 (3.7)	
No	28,275 (93.7)	4,530 (43.1)	
Missing	362 (1.2)	5,602 (53.3)	
**History of endometriosis, N (%)**			<0.001
Yes	1,419 (4.7)	321 (3.1)	
No	28,461 (94.3)	4,705 (44.7)	
Missing	312 (1.0)	5,494 (52.2)	
**Mental disease, N (%)**			<0.001
Yes	1,075 (3.6)	304 (2.9)	
No	28,740 (95.2)	4,574 (43.5)	
Missing	377 (1.2)	5,642 (53.6)	

Supplementary Table 1. Differences in the characteristics between participants who were analyzed and those who were excluded due to missing or clinically improbable data

**Variables**	**Study participants (N=30,192)**[1]	**Excluded participants (N=10,520)**[1]	***P*-value**[2]
**Breastfeeding experience, N (%)**			<0.001
Yes	23,919 (79.2)	5,855 (55.7)	
No	6,031 (20.0)	1,250 (11.9)	
Missing	242 (0.8)	3,415 (32.5)	
**Experience with oral contraceptives, N (%)**			<0.001
Yes	914 (3.0)	217 (2.1)	
No	28,395 (94.0)	6,773 (64.4)	
Missing	883 (2.9)	3,530 (33.6)	
**Experience with hormone replacement therapy, N (%)**			<0.001
Yes	1,971 (6.5)	417 (4.0)	
No	27,452 (90.9)	6,631 (63.0)	

Supplementary Table 1. Differences in the characteristics between participants who were analyzed and those who were excluded due to missing or clinically improbable data

**Variables**	**Study participants (N=30,192)[1]**	**Excluded participants (N=10,520)[1]**	***P*-value[2]**
Missing	769 (2.5)	3,472 (33.0)	
**Age <15 years at menarche, N (%)**			<0.001
<15 years	24,368 (80.7)	5,321 (50.6)	
≥15 years	5,545 (18.4)	1,972 (18.7)	
Missing	279 (0.9)	3,227 (30.7)	
Age **≥35 years at last delivery, N (%)**			<0.001
<35 years	23,148 (76.7)	6,014 (57.2)	
≥35 years	3,266 (10.8)	661 (6.3)	
Missing	3,778 (12.5)	3,845 (36.5)	
**Menstrual cycle, N (%)**			<0.001
Regular	23,344 (77.3)	5,113 (48.6)	
Irregular	5,322 (17.6)	1,280 (12.2)	
Missing	1,526 (5.1)	4,127 (39.2)	

Supplementary Table 1. Differences in the characteristics between participants who were analyzed and those who were excluded due to missing or clinically improbable data

Variables	Study participants (N=30,192)[1]	Excluded participants (N=10,520)[1]	*P*-value[2]
**History of HDP, N (%)**			<0.001
Yes	1,311 (4.3)	337 (3.2)	
No	28,881 (95.7)	4,673 (44.4)	
Missing	0 (0.0)	5,510 (52.4)	
**History of GDM, N (%)**			<0.001
No	30,108 (99.7)	4,763 (45.3)	
Yes	84 (0.3)	14 (0.1)	
Missing	0 (0.0)	5,743 (54.6)	
**Family history of glomerulonephritis, N (%)**			<0.001
No	29,679 (98.3)	4,773 (45.4)	
Yes	145 (0.5)	29 (0.3)	
Missing	368 (1.2)	5,718 (54.4)	

Supplementary Table 1. Differences in the characteristics between participants who were analyzed and those who were excluded due to missing or clinically improbable data

Variables	Study participants (N=30,192)[1]	Excluded participants (N=10,520)[1]	*P*-value[2]
**Family history of kidney dialysis, N (%)**			<0.001
No	29,489 (97.7)	4,754 (45.2)	
Yes	333 (1.1)	72 (0.7)	
Missing	370 (1.2)	5,694 (54.1)	
**Family history of hypertension, N (%)**			<0.001
Yes	11,482 (38.0)	2,651 (25.2)	
No	18,537 (61.4)	4,422 (42.0)	
Missing	173 (0.6)	3,447 (32.8)	
**Family history of type 2 diabetes, N (%)**			<0.001
Yes	3,370 (11.2)	725 (6.9)	

Supplementary Table 1. Differences in the characteristics between participants who were analyzed and those who were excluded due to missing or clinically improbable data

Variables	Study participants (N=30,192)[1]	Excluded participants (N=10,520)[1]	*P*-value[2]
No	26,485 (87.7)	4,666 (44.4)	
Missing	337 (1.1)	5,129 (48.8)	
**Marital status, N (%)**			<0.001
Married	23,545 (78.0)	6,371 (60.6)	
Unmarried	1,908 (6.3)	348 (3.3)	
Divorced	1,375 (4.6)	400 (3.8)	
Widowed	3,192 (10.6)	1,117 (10.6)	
Missing	172 (0.6)	2,284 (21.7)	
**Highest level of education, N (%)**			<0.001
Low	5,511 (18.3)	2,572 (24.4)	
Medium	20,207 (66.9)	5,120 (48.7)	
High	4,171 (13.8)	735 (7.0)	
Missing	303 (1.0)	2,093 (19.9)	

Supplementary Table 1. Differences in the characteristics between participants who were analyzed and those who were excluded due to missing or clinically improbable data

**Variables**	**Study participants (N=30,192)[1]**	**Excluded participants (N=10,520)[1]**	***P*-value[2]**
**Frequency of breakfast, N (%)**			<0.001
Every day	27,021 (89.5)	7,642 (72.6)	
Skipping	2,702 (8.9)	756 (7.2)	
Missing	469 (1.6)	2,122 (20.2)	
**Sleeping time, N (%)**			<0.001
<7 h	22,443 (74.3)	6,253 (59.4)	
≥7 and ≤8 h	5,933 (19.7)	1,794 (17.1)	
≥8 h	1,787 (5.9)	588 (5.6)	
Missing	29 (0.1)	1,885 (17.9)	
**Nap time, N (%)**			<0.001
Not taking a nap	18,801 (62.3)	5,101 (48.5)	
<1 h/day	9,078 (30.1)	2,758 (26.2)	
≥1 h/day	2,177 (7.2)	696 (6.6)	

Supplementary Table 1. Differences in the characteristics between participants who were analyzed and those who were excluded due to missing or clinically improbable data

**Variables**	**Study participants (N=30,192)[1]**	**Excluded participants (N=10,520)[1]**	***P*-value[2]**
Missing	136 (0.5)	1,965 (18.7)	
**Number of relocations after the GEJE, N (%)**			<0.001
0	22,808 (75.5)	5,585 (53.1)	
1	2,233 (7.4)	769 (7.3)	
2	1,503 (5.0)	630 (6.0)	
3	1,275 (4.2)	477 (4.5)	
≥4	727 (2.4)	246 (2.3)	
Missing	1,646 (5.5)	2,813 (26.7)	
**Year, N (%)**			<0.001
2013	5,061 (16.8)	5,261 (50.0)	
2014	13,605 (45.1)	2,980 (28.3)	
2015	11,526 (38.2)	2,272 (21.6)	

Supplementary Table 1. Differences in the characteristics between participants who were analyzed and those who were excluded due to missing or clinically improbable data

**Variables**	**Study participants (N=30,192)**[1]	**Excluded participants (N=10,520)**[1]	***P*-value**[2]
**Prefecture, N (%)**			<0.001
Miyagi	16,637 (55.1)	7,582 (72.1)	
Iwate	13,555 (44.9)	2,938 (27.9)	
**Menopausal status, N (%)**			<0.001
Premenopausal	6,605 (21.9)	1,341 (12.7)	
Postmenopausal	23,587 (78.1)	5,135 (48.8)	
Missing	0 (0.0)	4,044 (38.4)	
**Menopausal age, N (%)**			<0.001
Premenopause	6,605 (21.9)	1,341 (12.7)	
Premature menopause (age at menopause <40 years)	909 (3.0)	232 (2.2)	
Postmenopause (age at menopause ≥40 years)	22,134 (73.3)	4,730 (45.0)	

Supplementary Table 1. Differences in the characteristics between participants who were analyzed and those who were excluded due to missing or clinically improbable data

**Variables**	**Study participants (N=30,192)[1]**	**Excluded participants (N=10,520)[1]**	***P*-value[2]**
Missing	544 (1.8)	4,217 (40.1)	
**Reasons of menopause, N (%)**			<0.001
Premenopause	6,605 (21.9)	1,321 (12.6)	
Natural menopause	19,083 (63.2)	3,452 (32.8)	
Menopause due to surgery of uterus and/or ovary	3,439 (11.4)	710 (6.7)	
Other reasons	762 (2.5)	150 (1.4)	
Missing	303 (1.0)	4,887 (46.5)	

[1]Continuous and categorical variables are shown as means (standard deviations) and numbers (percentages), respectively.

[2]Pearson's Chi-squared test; Student's t-test

Abbreviations: BMI, body mass index; γ-GTP, γ-Glutamyl transpeptidase; SBP, systolic blood pressure; DBP, diastolic blood pressure; HDP, hypertensive disorders of pregnancy; GDM, gestational diabetes mellitus; GEJE, Great East Japan Earthquake.

Supplementary Table 2. Association between parity and urine ACR ≥300 mg/gCre in postmenopausal multiparous women

**Model**	**Parity**				***P*-value for trend**	**History of HDP**	**History of GDM**	**BMI at 20-years-old, per 1-SD increase**	**Current BMI, per 1-SD increase**
	**1 (N=2,083)**	**2 (N=11,467)**	**3 (N=7,169)**	**≥4 (N=1,220)**					
**Cases with urine ACR ≥300 mg/gCre (%)**	11 (0.5)	71 (0.6)	61 (0.9)	5 (0.4)	0.29	-	-	-	-
**Model 1** Adjusted OR	Reference	1.123 (0.5942.124)	1.603 (0.842–3.053)	0.771 (0.267–2.224)	0.25	-	-	-	-

(95% CI)									
**Model 2**	Reference	1.143	1.395	0.937	0.13	-	-	-	-
Adjusted OR		(0.827–1.579)	(1.003–1.941)	(0.546–1.608)					
(95% CI)									
**Model 3**	Reference	1.135	1.359	0.916	0.19	-	-	-	-
Adjusted OR		(0.821–1.568)	(0.977–1.890)	(0.534–1.571)					
(95% CI)									
**Model 4**	Reference	1.135	1.363	0.917	0.18	1.473	1.411	-	-
Adjusted OR		(0.821–1.568)	(0.979–1.898)	(0.534–1.574)		(1.106–	(0.482–		
(95% CI)						1.961)	4.125)		
**Model 5**	Reference	1.135	1.364	0.918	0.18	1.474	1.411	0.991	-

Adjusted OR (95% CI)		(0.821–1.568)	(0.980–1.899)	(0.534–1.575)		(1.107–1.963)	(0.483–4.128)	(0.841–1.167)	
**Model 6**	Reference	1.147	1.337	0.875	0.33	1.380	1.498	-	1.396
Adjusted OR (95% CI)		(0.829–1.587)	(0.960–1.862)	(0.509–1.503)		(1.032–1.845)	(0.514–4.362)		(1.255–1.553)

Model 1: Adjusting for age.

Model 2: Model 1 variables in addition to height, physical activity, marital status, smoking status, alcohol consumption, own birth weight, highest educational level, family history of hypertension, family history of type 2 diabetes mellitus, family history of glomerulonephritis, breastfeeding experience, oral contraceptive use, hormone replacement therapy use, thyroid dysfunction, endometriosis, mental disease, menstrual cycle, age at menarche (<15 years or ≥15 years), age at last delivery (<35 years or ≥35 years), menopause age (<40 years or ≥40 years), sleeping time, nap time, year of study participation,

Prefecture (Miyagi or Iwate), and number of relocations after the GEJE.

Model 3: Model 2 variables, γ-GTP (<50 or ≥50 IU/l), and estimated 24 h NaCl and K intakes.

Model 4: Model 3 variables and a history of HDP and GDM.

Model 5: Model 4 variables and BMI at 20 years old as per 1-SD increase.

Model 6: Model 4 variables and current BMI as per 1-SD increase.

Abbreviations: ACR, albumin-to-creatinine ratio; BMI, body mass index; CI, confidence interval; HDP, hypertensive disorders of pregnancy; GDM, gestational diabetes mellitus; GEJE, Great East Japan Earthquake; γ-GTP, γ-glutamyl transpeptidase; OR, odds ratio; SD, standard deviation; NaCl, sodium chloride; K, potassium.

Supplementary Table 3. Association between parity and urine ACR ≥300 mg/gCre in all postmenopausal women

**Model**	**Parity**					***P*-value for trend**	**BMI at 20-years-old, per 1-SD increase**	**Current BMI, per 1-SD increase**
	**0 (N=1,648)**	**1 (N=2,083)**	**2 (N=11,467)**	**3 (N=7,169)**	**≥4 (N=1,220)**			
**Cases with urine ACR ≥300 mg/gCre (%)**	9 (0.6)	11 (0.5)	71 (0.6)	61 (0.9)	5 (0.4)	0.23	-	-
**Model 1** Adjusted OR (95% CI)	1.162 (0.479–2.815)	Reference	1.122 (0.593–2.122)	1.603 (0.842–3.054)	0.771 (0.267–2.225)	0.30	-	-
**Model 2**	1.150	Reference	1.101	1.300	0.858	0.42	-	-

Adjusted OR	(0.717–		(0.798–	(0.938–	(0.503–			
(95% CI)	1.845)		1.518)	1.802)	1.465)			
**Model 3**	1.161	Reference	1.094	1.271	0.842	0.55	-	-
Adjusted OR	(0.724–		(0.793–	(0.917–	(0.494–			
(95% CI)	1.861)		1.509)	1.762)	1.438)			
**Model 4**	1.161	Reference	1.095	1.269	0.841	0.57	1.045	-
Adjusted OR	(0.724–		(0.794–	(0.915–	(0.493–		(0.909–	
(95% CI)	1.861)		1.510)	1.759)	1.435)		1.201)	
**Model 5**	1.189	Reference	1.115	1.254	0.806	0.81	-	1.397
Adjusted OR	(0.741–		(0.807–	(0.904–	(0.472–			(1.260–
(95% CI)	1.906)		1.539)	1.741)	1.378)			1.549)

Model 1: Adjusting for age.

Model 2: Model 1 variables in addition to height, physical activity, marital status, smoking status, alcohol consumption, own birth weight, highest educational level, family history of hypertension, family history of type 2 diabetes mellitus, family history of glomerulonephritis, oral contraceptive use, hormone replacement therapy use, thyroid dysfunction, endometriosis, mental disease, menstrual cycle, age at menarche (<15 years or ≥15 years), menopause age (<40 years or ≥40 years), sleeping time, nap time, year of study participation, prefecture (Miyagi or Iwate), and number of relocations after the GEJE.

Model 3: Model 2 variables, γ-GTP (<50 IU/l or ≥50 IU/l), and estimated 24 h NaCl and K intakes.

Model 4: Model 3 variables and BMI at 20 years old, as per 1-SD increase.

Model 5: Model 3 variables and current BMI, as per 1-SD increase.

Abbreviations: ACR, albumin-to-creatinine ratio; BMI, body mass index; CI, confidence interval; GEJE, Great East Japan Earthquake; γ-GTP, γ-glutamyl transpeptidase; OR, odds ratio; SD, standard deviation; NaCl, sodium chloride; K, potassium.

Supplementary Figure 1

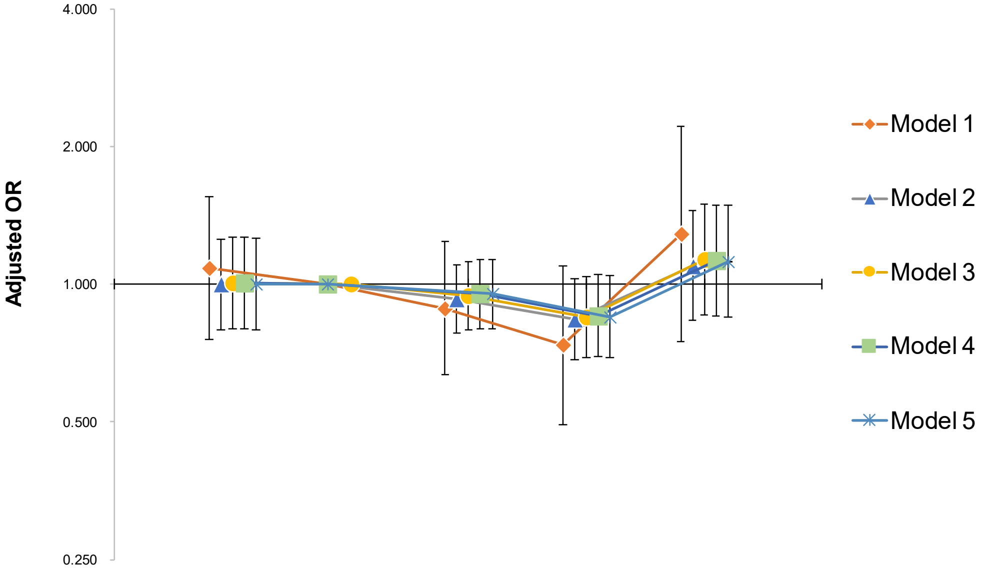

Model	Parity					*P*-value for trend	BMI at 20-years-old, per 1-SD increase†	Current BMI, per 1-SD increase*
	0 (N=1,599)	1 (N=1,110)	2 (N=2,448)	3 (N=1,186)	≥4 (N=262)			
Cases of CKD (%)	77 (4.8)	53 (4.8)	119 (4.9)	52 (4.4)	20 (7.6)	0.57	-	-
**Model 1** Adjusted OR (95% CI)	1.086 (0.757-1.557)	Reference	0.887 (0.635-1.240)	0.736 (0.494-1.095)	1.288 (0.751-2.210)	0.19	-	-
**Model 2** Adjusted OR (95% CI)	0.999 (0.795-1.256)	Reference	0.929 (0.783-1.101)	0.840 (0.686-1.030)	1.098 (0.834-1.447)	0.40	-	-
**Model 3** Adjusted OR (95% CI)	1.009 (0.801-1.270)	Reference	0.943 (0.794-1.120)	0.849 (0.691-1.042)	1.132 (0.857-1.497)	0.48	-	-
**Model 4** Adjusted OR (95% CI)	1.008 (0.800-1.270)	Reference	0.952 (0.801-1.131)	0.854 (0.695-1.049)	1.128 (0.853-1.492)	0.51	1.208 (1.089-1.339)	-
**Model 5** Adjusted OR (95% CI)	1.002 (0.795-1.264)	Reference	0.952 (0.801-1.131)	0.850 (0.692-1.044)	1.124 (0.850-1.487)	0.51	-	1.216 (1.136-1.302)

Supplementary Figure 2

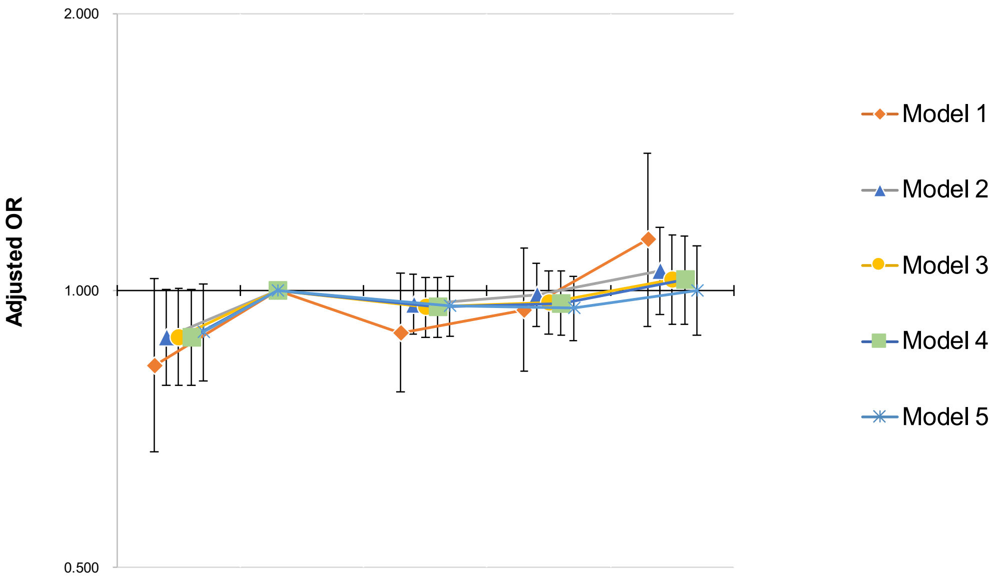

Model	Parity					*P*-value for trend	BMI at 20-years-old, per 1-SD increase†	Current BMI, per 1-SD increase*
	0 (N=1,648)	1 (N=2,083)	2 (N=11,467)	3 (N=7,169)	≥4 (N=1,220)			
**Cases of CKD (%)**	148 (9.0)	238 (11.4)	1,236 (10.8)	794 (11.1)	157 (12.9)	0.011	-	-
**Model 1** Adjusted OR (95% CI)	0.829 (0.667-1.031)	Reference	0.900 (0.776-1.044)	0.953 (0.816-1.112)	1.136 (0.915-1.411)	0.051	-	-
**Model 2** Adjusted OR (95% CI)	0.889 (0.788-1.004)	Reference	0.966 (0.896-1.041)	0.990 (0.915-1.071)	1.050 (0.941-1.172)	0.043	-	-
**Model 3** Adjusted OR (95% CI)	0.890 (0.788-1.005)	Reference	0.959 (0.889-1.034)	0.970 (0.896-1.050)	1.028 (0.920-1.148)	0.18	-	-
**Model 4** Adjusted OR (95% CI)	0.890 (0.788-1.004)	Reference	0.959 (0.889-1.034)	0.969 (0.895-1.049)	1.027 (0.919-1.147)	0.19	1.015 (0.973-1.058)	-
**Model 5** Adjusted OR (95% CI)	0.901 (0.798-1.017)	Reference	0.962 (0.891-1.037)	0.956 (0.883-1.035)	0.999 (0.894-1.117)	0.20	-	1.198 (1.162-1.235)

Supplementary Figure 3

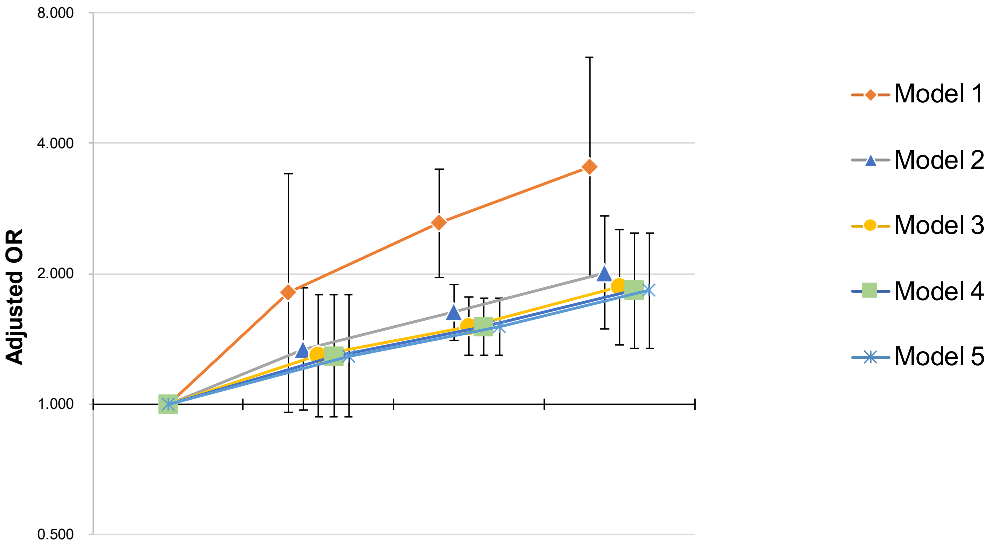

Obesity	No		Yes				
Clinical history of HDP	No (N=3,843)	Yes (N=175)	No (N=873)	Yes (N=115)	*P*-value for interaction	History of GDM	BMI at 20-years-old, per 1-SD increase†
Cases of CKD (%)	136 (3.5)	11 (6.3)	83 (9.5)	14 (12.2)	-	-	-
**Model 1** Adjusted OR (95% CI)	Reference	1.805 (0.956-3.409)	2.616 (1.964-3.485)	3.521 (1.957-6.335)	<0.0001	-	-
**Model 2** Adjusted OR (95% CI)	Reference	1.340 (0.970-1.851)	1.630 (1.405-1.891)	2.012 (1.486-2.723)	0.72	-	-
**Model 3** Adjusted OR (95% CI)	Reference	1.293 (0.934-1.789)	1.513 (1.300-1.762)	1.861 (1.369-2.529)	0.83	-	-
**Model 4** Adjusted OR (95% CI)	Reference	1.290 (0.932-1.785)	1.512 (1.299-1.760)	1.827 (1.342-2.489)	0.78	1.323 (0.810-2.160)	-
**Model 5** Adjusted OR (95% CI)	Reference	1.289 (0.931-1.784)	1.480 (1.257-1.742)	1.773 (1.287-2.442)	0.75	1.323 (0.811-2.160)	1.053 (0.915-1.212)

Supplementary Figure 4

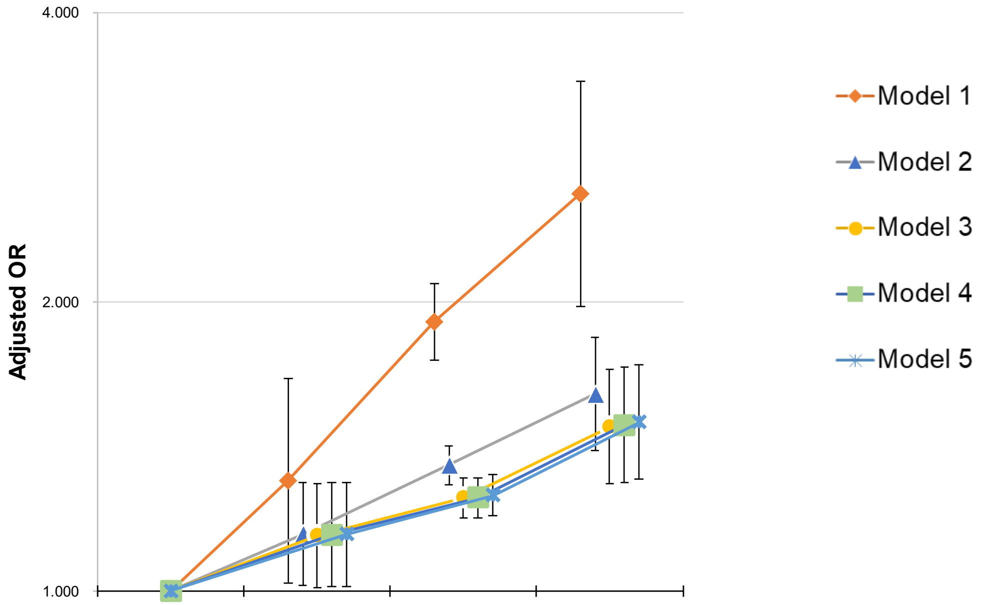

Obesity	No		Yes		*P*-value for interaction	History of GDM	BMI at 20-years-old, per 1-SD increase†
**Clinical history of HDP**	**No (N=15,640)**	**Yes (N=672)**	**No (N=5,278)**	**Yes (N=349)**			
**Cases of CKD (%)**	1,417 (9.1)	76 (11.3)	862 (16.3)	70 (20.1)	-	-	-
**Model 1** Adjusted OR (95% CI)	Reference	1.303 (1.019-1.666)	1.906 (1.739-2.088)	2.592 (1.980-3.392)	0.02	-	-
**Model 2** Adjusted OR (95% CI)	Reference	1.146 (1.013-1.297)	1.352 (1.290-1.416)	1.603 (1.400-1.836)	0.72	-	-
**Model 3** Adjusted OR (95% CI)	Reference	1.142 (1.008-1.293)	1.251 (1.192-1.313)	1.483 (1.293-1.702)	0.69	-	-
**Model 4** Adjusted OR (95% CI)	Reference	1.144 (1.010-1.296)	1.251 (1.192-1.313)	1.489 (1.297-1.709)	0.68	0.791 (0.428-1.463)	-
**Model 5** Adjusted OR (95% CI)	Reference	1.146 (1.010-1.296)	1.258 (1.198-1.322)	1.499 (1.306-1.722)	0.68	0.793 (0.430-1.466)	0.973 (0.930-1.018)
